# Supplementary material for: Systems-level analysis predicts no autotrophy-linked protein-RNA interactions in Clostridium autoethanogenum
Source: Nat Commun. 2026 Jul 8;17:8435. doi: 10.1038/s41467-026-75309-w (PMC13478517; doi:10.1038/s41467-026-75309-w)
Supplement: Supplementary file 1 [file 41467_2026_75309_MOESM1_ESM.pdf]

<u>Supplementary Information</u>

Systems-level analysis predicts no autotrophy-linked

protein-RNA interactions in *Clostridium autoethanogenum*

Angela Re, Gianfranco Michele Maria Politano, Kristina Reinmets, Alfredo Benso,

Laurence Girbal, Muriel Cocaign-Bousquet, Kaspar Valgepea

Supplementary Methods

Composition of the chemically defined yeast extract-free medium (per liter) used in cultivations

0.5 g $MgCl_2 \cdot 6H_2O$, 0.2 g NaCl, 0.1 g $CaCl_2$, 2.65 g $NaH_2PO_4 \cdot 2H_2O$, 0.5 g KCl, 2.5 g NH_4Cl, 0.017 g $FeCl_3 \cdot 6H_2O$, 0.5 g cysteine-HCl·H_20, 1 mL of 2 g/L resazurin, 10 mL trace metal solution (TMS) and 10 mL B-vitamin solution. The TMS contained per liter: 1.5 g nitrilotriacetic acid, 3 g $MgSO_4 \cdot 7H_2O$, 0.5 g $MnSO_4 \cdot H_2O$, 1 g NaCl, 0.667 g $FeSO_4 \cdot 7H_2O$, 0.2 g $CoCl_2 \cdot 6H_2O$, 0.2 g $ZnSO_4 \cdot 7H_2O$, 0.02 g $CuCl_2 \cdot 2H_2O$, 0.014 g $Al_2(SO_4)_3 \cdot 18H_2O$, 0.3 g H_3BO_3, 0.03 g $NaMoO_4 \cdot 2H_2O$, 0.02 g Na_2SeO_3, 0.02 g $NiCl_2 \cdot 6H_2O$ and 0.02 g $Na_2WO_4 \cdot 2H_2O$. The B-vitamin solution contained per liter: 20 mg biotin, 20 mg folic acid, 10 mg pyridoxine hydrochloride, 50 mg thiamine-HCl, 50 mg riboflavin, 50 mg nicotinic acid, 50 mg calcium pantothenate, 50 mg vitamin B_{12}, 50 mg 4-aminobenzoic acid, and 50 mg thioctic acid.

Cross-comparison of top 5% gene sets across the three metrics used to evaluate translational regulation (TL-STATUS, TL-PROFILE, and POLY-FRACTIONS)

We used two approaches to compare the distributions of gene ranks across the three metrics we used to evaluate translational regulation.

Approach A: Here, we normalized gene ranks to the range [0, 100] according to each of the metrics that we used to evaluate translational regulation (TL-STATUS, TL-PROFILE, and POLY-FRACTIONS). Firstly, we determined the normalized ranks that the top 5% ranking genes by a certain metric obtained by using another metric. Secondly, we evaluated if this rank distribution is significantly different from a rank distribution obtained by random sampling of gene ranks without replacement from a uniform distribution in the interval [0,100]. For this, we used both the Mann-Whitney (MW) test, which detects differences in central tendency, and the Kolmogorov-Smirnov (KS) test, which is sensitive to any difference in the distribution shapes. Since the statistical significance of the test result can vary according to the randomly sampled rank distribution, we repeated both tests 10^6 times to assess their range of variability. We carried out this procedure for each pair of the three metrics. Comparing the ranges of obtained p-values (see Supplementary Fig. 5a below) showed that the top 5% gene sets from any of the three metrics are ranked statistically significantly higher than randomly sampled gene rankings when evaluating their rankings in the ranking obtained by another metric (significance threshold p-value 0.05; i.e., $-\log_{10}$(p-value) ~1.3).

Approach B: Identical to Approach A, we normalized gene ranks to the range [0, 100] according to each of the metrics that we used to evaluate translational regulation (TL-STATUS, TL-PROFILE, and POLY-FRACTIONS). Firstly, we determined the normalized ranks that the top 5% ranking genes by a certain metric obtain by using another metric. Secondly, we considered the median value of these ranks for each metric as the test statistic and evaluated if it significantly differs from random expectations. For this, we constructed the null distribution of the test statistic by computing its value for each of the 10^6 random rank distributions that were obtained by random sampling a number of ranks equal to the number of top 5% ranking genes from a uniform distribution on the interval [0, 100]. We carried out this procedure for each pair of the three metrics. The empirical p-value was then obtained as the fraction of cases that produce a test statistic at least as low as that calculated for experimental data. This analysis showed that the median rank of top 5% ranking genes of any of the three metrics is statistically significantly lower than that of randomly sampled genes in the ranking obtained by another metric (see Supplementary Fig. 5b below; p-value < 10^{-6}).

Supplementary Figures

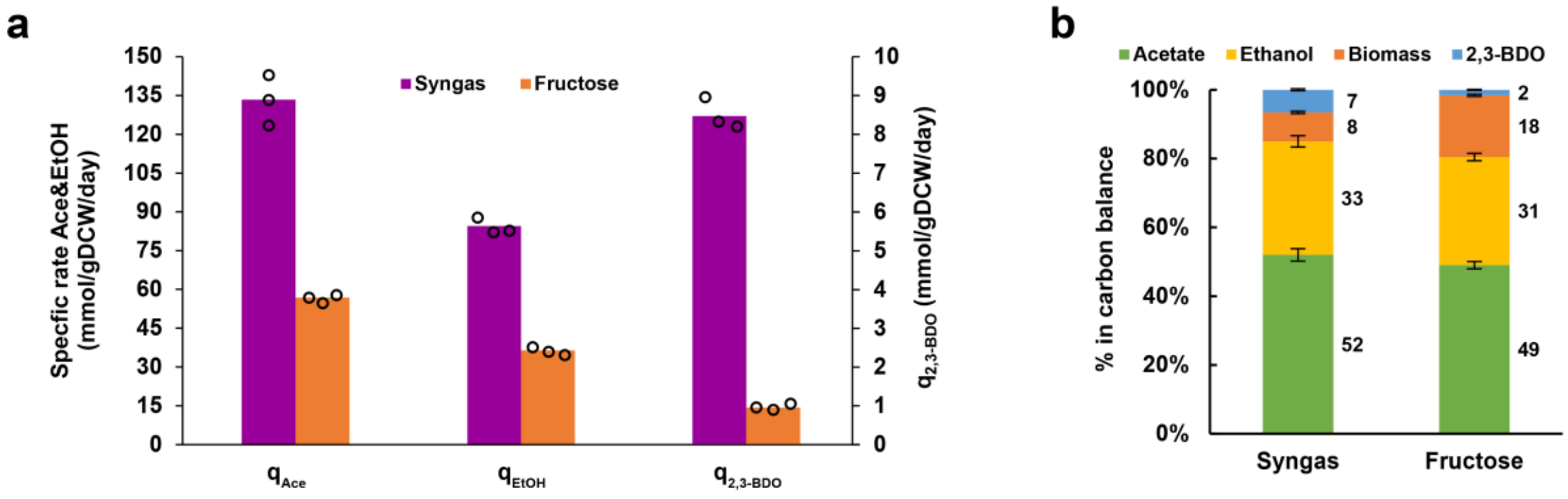

Supplementary Fig. 1. **Specific by-product production rates and carbon balances in *C. autoethanogenum* syngas and fructose chemostats. a** Specific by-product production rates (mmol per gram of dry cell weight per day). Ace, acetate; EtOH, ethanol; 2,3-BDO, 2,3-butanediol. **b** Carbon balances. Carbon recoveries were normalized to 100% to have a fair comparison of carbon distributions between conditions. Bars show average ± standard deviation between n=3 independent chemostat cultures (shown for better readability). Source data are provided as a Source Data file.

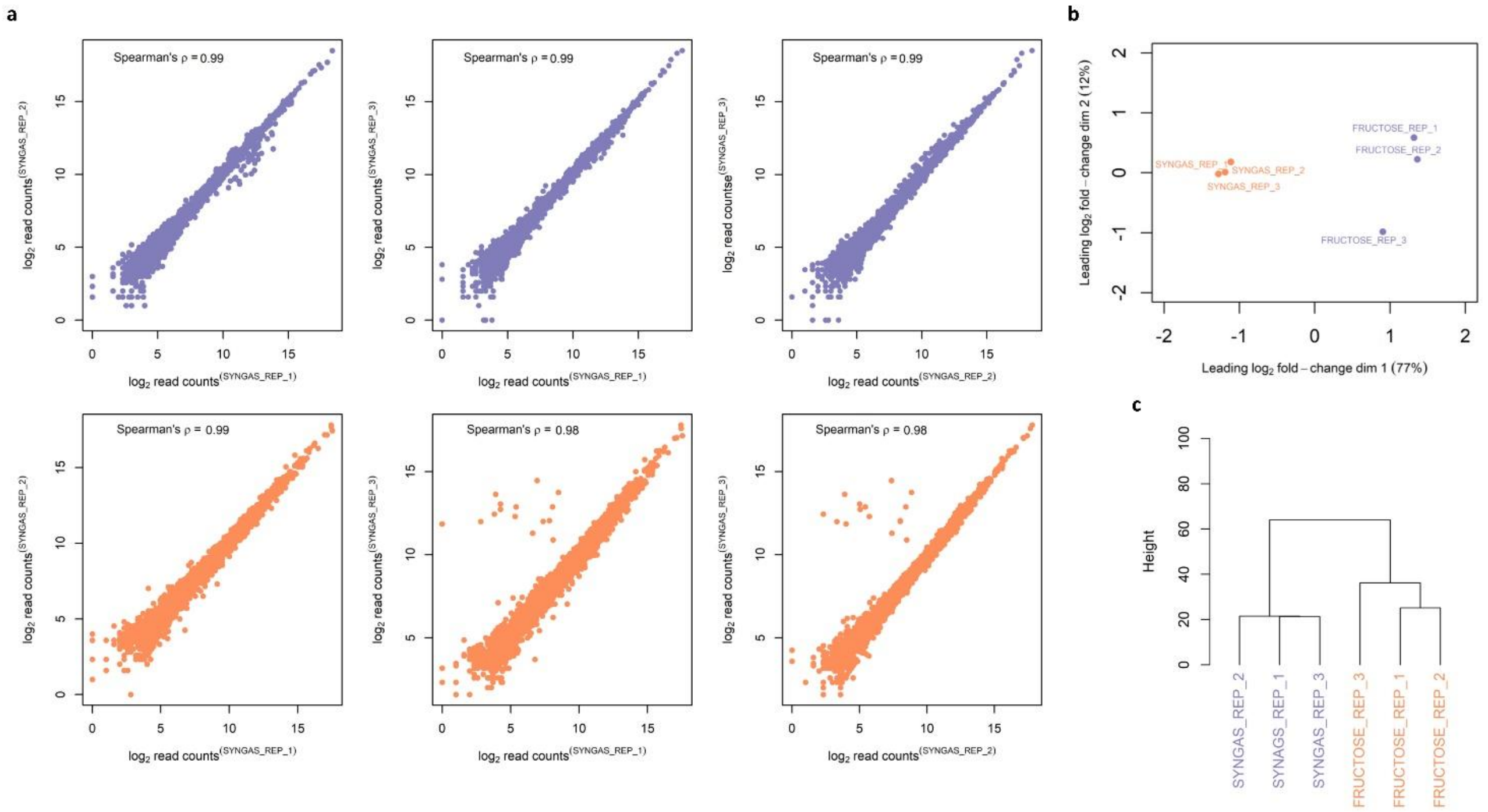

Supplementary Fig. 2. **Reproducibility and clustering of RNA-seq data across *C. autoethanogenum* syngas and fructose chemostats. a** Correlation of normalized $\log_2$ RNA-seq read counts between syngas and fructose biological triplicate chemostat cultures. **b** Clustering of syngas and fructose biological triplicate chemostat cultures on a multidimensional scaling plot. Distances represent leading $\log_2$ fold-changes that are the root-mean-square average of the top 500 largest $\log_2$ fold-changes between any two samples. **c** Clustering of syngas and fructose biological triplicate chemostat cultures on a dendrogram based on normalized $\log_2$ RNA-seq read counts.

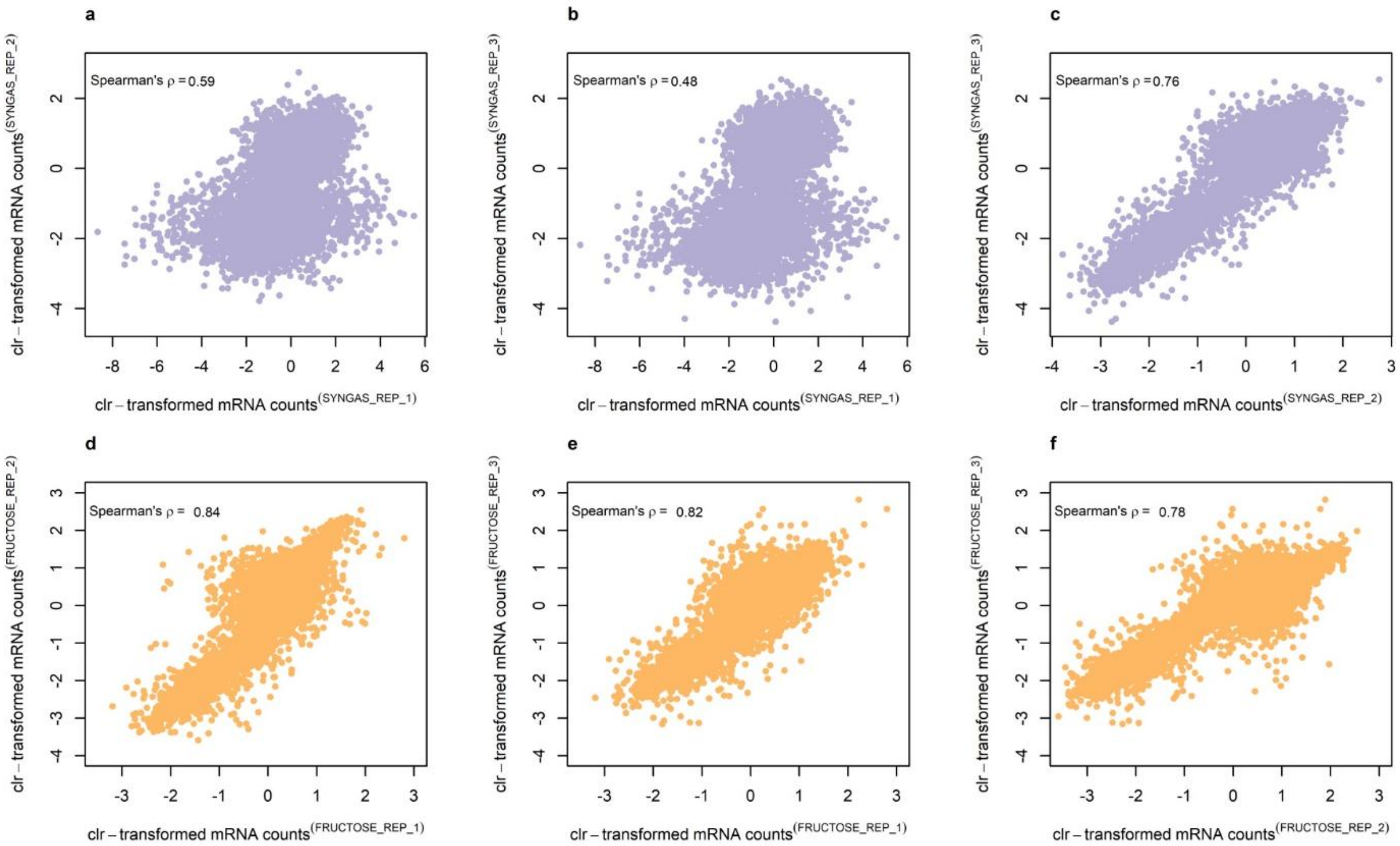

Supplementary Fig. 3. Reproducibility of polysome-seq data across *C. autoethanogenum* syngas and fructose chemostats. a-c Correlation of centered-ln-ratio transformed (clr) mRNA counts per polysomal gradient fraction from total counts between biological triplicate syngas chemostat cultures. **d-f** Correlation of clr mRNA counts per polysomal gradient fractions from total counts between biological triplicate fructose chemostat cultures.

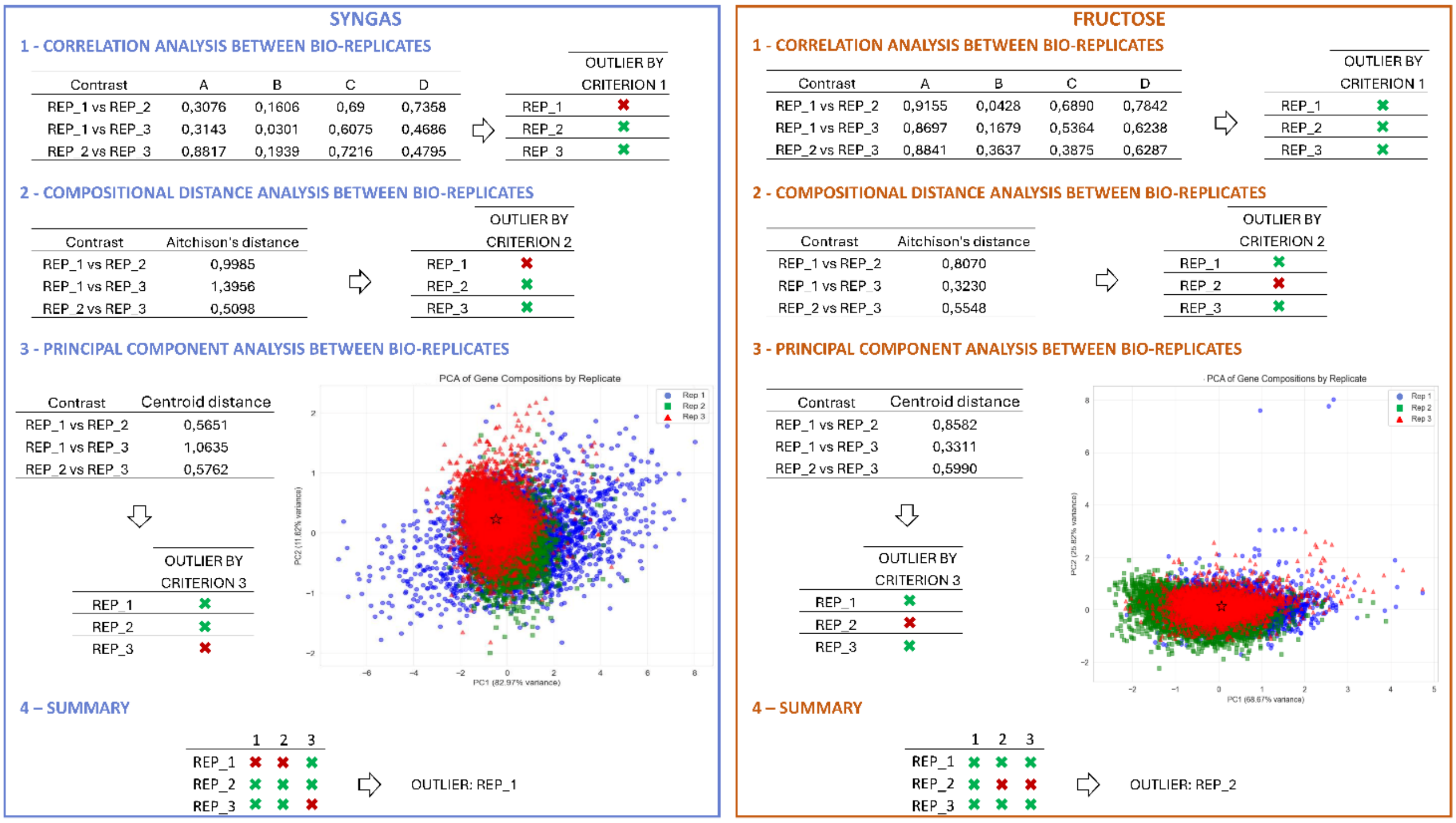

Supplementary Fig. 4. Outlier detection in polysome-seq data across bio-replicate *C. autoethanogenum* syngas and fructose chemostats. Consistency between bio-replicates within both chemostat datasets was assessed by three complementary methods: 1) Correlation analysis of mRNA counts per polysomal gradient fractions from total counts between bio-replicates by Spearman's rank correlation coefficient ρ. A bio-replicate was determined as an outlier if it showed the highest number of poorly correlated polysomal fractions; a fraction was considered poorly correlated if its data correlated poorly ($\rho < 0.7$) with the other bio-replicates which in turn correlated between each other with $\rho > 0.7$. 2) Compositional distance analysis by first calculating the average of all mRNA counts for each fraction and bio-replicate, then centered-ln-ratio transforming the averages, and lastly computing the Aitchison's distance between each pair of bio-replicates. A bio-replicate was determined as an outlier if it showed the maximal average Aitchison's distance from the other bio-replicates that was greater than the median distance observed across syngas and fructose. 3) Principal component analysis (PCA) by first collating all mRNA counts for each fraction and bio-replicate as a concatenated dataset, then centered-ln-ratio transforming gene compositional data and lastly performing PCA to compute the average distances between the centroids of each pair of bio-replicates. A bio-replicate was determined as an outlier if its centroid showed the maximal average distance from the centroids of the other bio-replicates that was greater than the median distance observed across syngas and fructose.

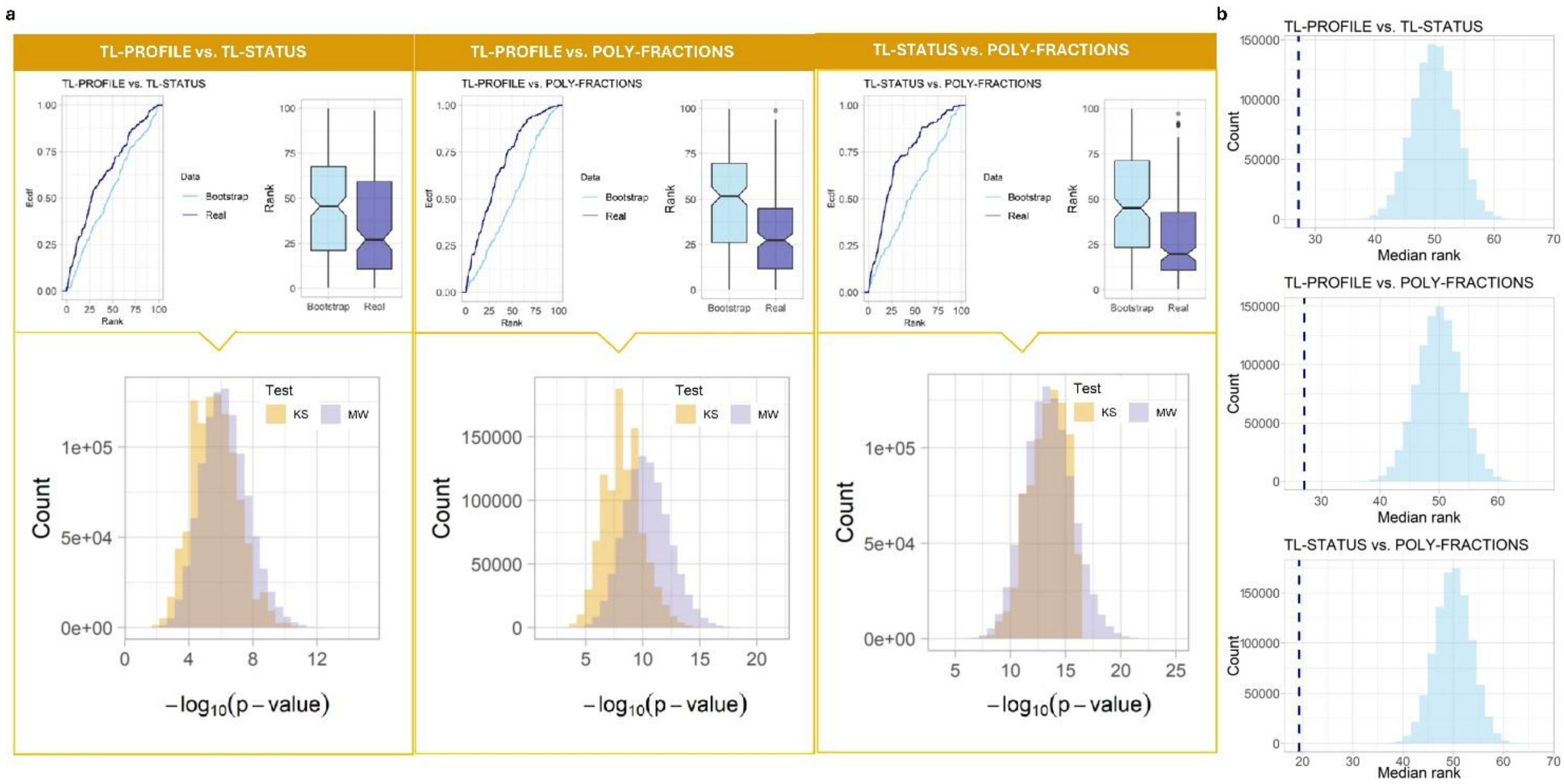

Supplementary Fig. 5. **Cross-comparison of top 5% gene sets across the three metrics used to evaluate translational regulation. a** Analysis results from Approach A (see Supplementary Methods text for details). Top row shows differences in the empirical cumulative distribution function and central tendency between the ranks of the top 5% gene set ('Real') and randomly sampled ranks ('Bootstrap'). Bottom row shows -log$_{10}$(p-value) distributions. Ecdf, empirical cumulative distribution function; KS, Kolmogorov-Smirnov test; MW, Mann-Whitney test. **b** Analysis results from Approach B (see Supplementary Methods text for details). Median rank null distribution from random sampling is shown in light blue and median normalized rank from experimental data is shown as dashed dark blue line.

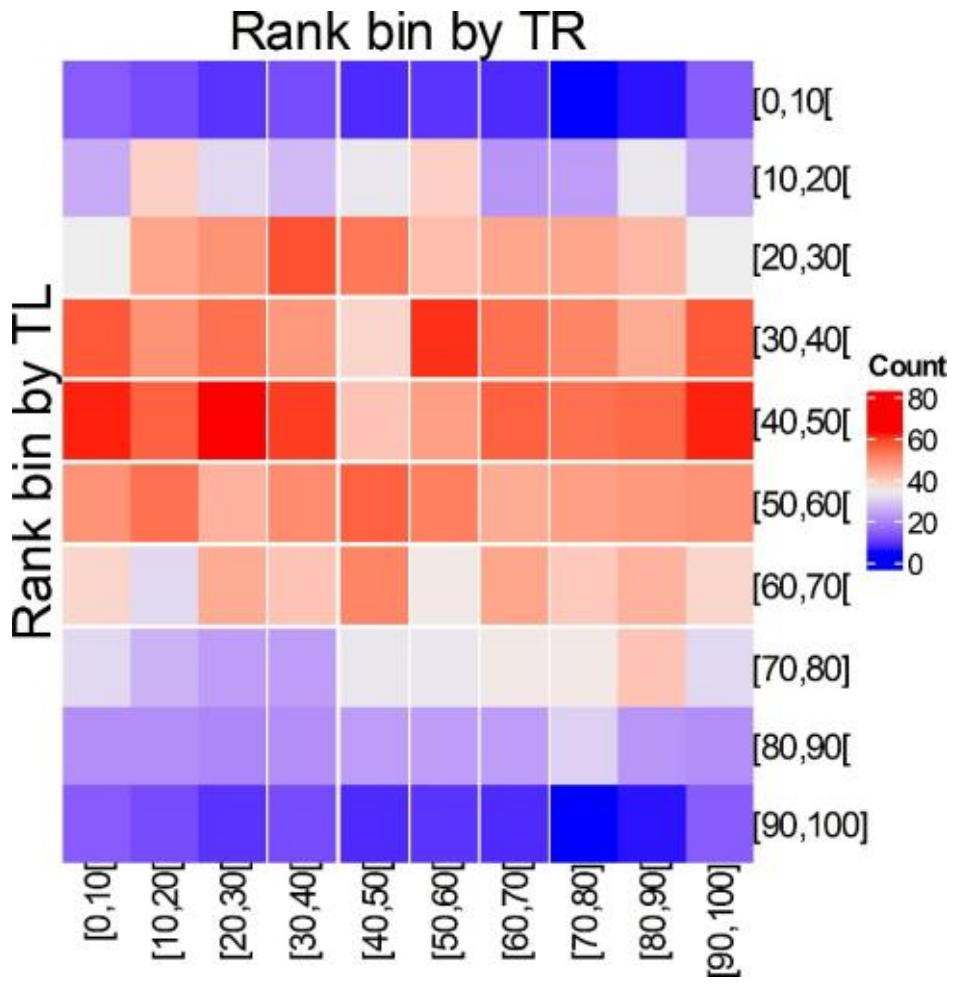

Supplementary Fig. 6. Near lack of agreement between highly ranking gene sets in differential transcription and differential translation analyses. The heatmap displays gene counts by bins of ranks produced by differential transcription (TR) and differential translation (TL) analysis. Ranks are scaled to the interval [0, 100].

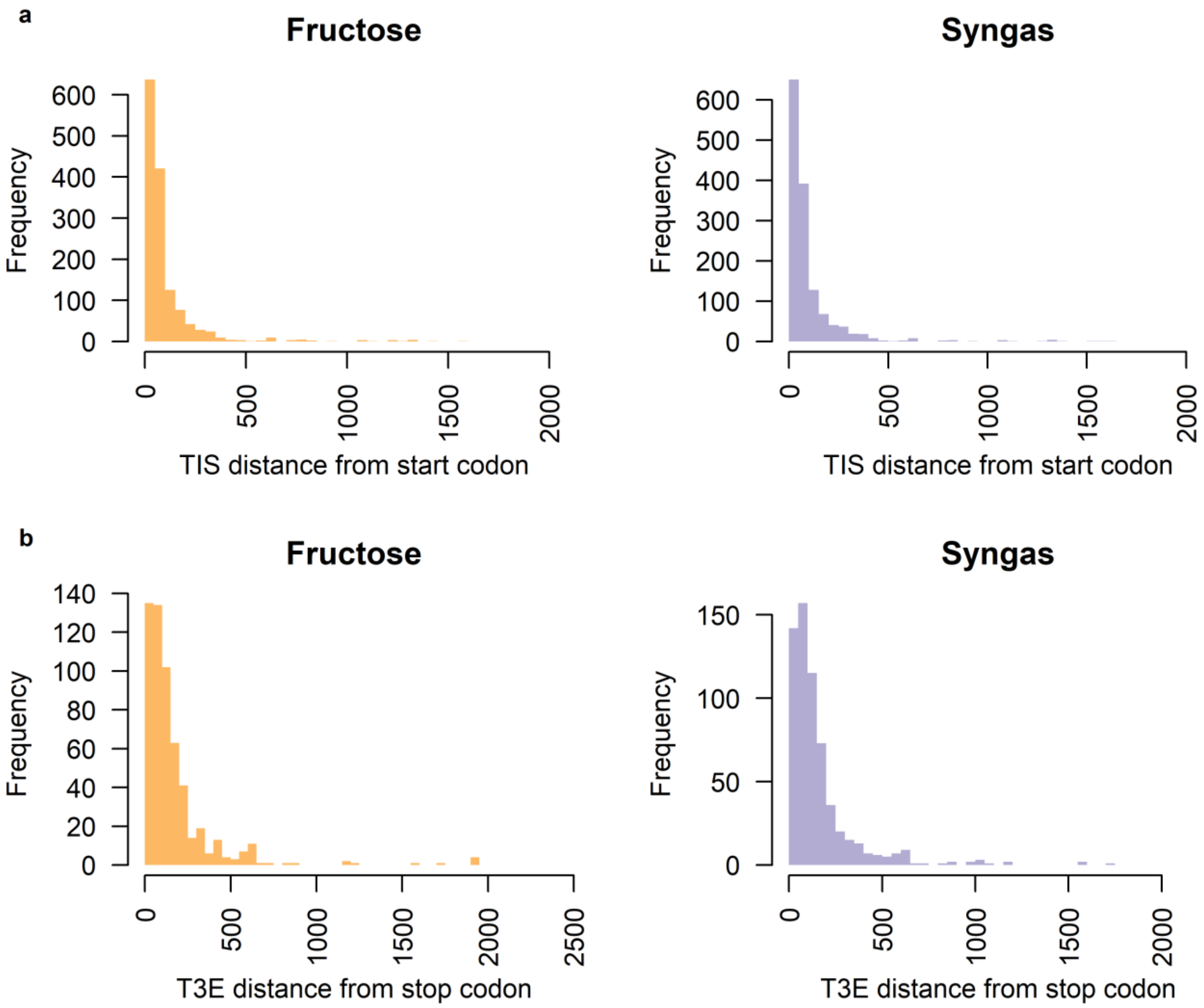

Supplementary Fig. 7. **Distribution of 5’UTR and 3’UTR lengths in *C. autoethanogenum* syngas and fructose chemostats. a** 5’UTR length as distance of transcription initiation site (TIS) from start codon of coding DNA sequence. **b** 3’UTR length as distance of transcription 3’ end (T3E) from stop codon of coding DNA sequence. Bars are aggregate of merged data from n=3 independent chemostat cultures.

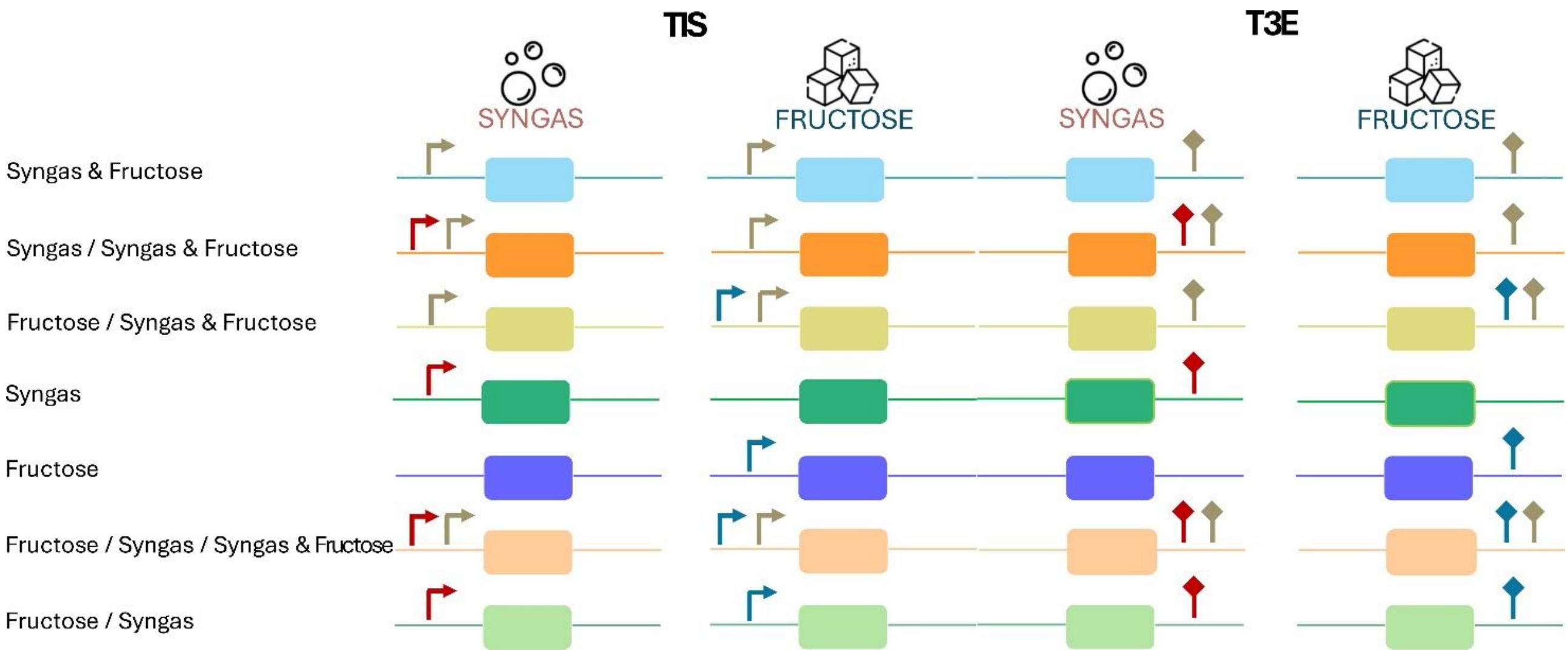

Supplementary Fig. 8. Detected gene-specific TIS and T3E profiles for syngas and fructose *C. autoethanogenum* chemostats. Scheme supports Fig. 5e and 5f. TISs or T3Es present uniquely in syngas are colored red, TISs or T3Es present uniquely in fructose are blue, TISs or T3Es shared by fructose and syngas are brown. Syngas & Fructose, site(s) shared between two conditions; Syngas / Syngas & Fructose, site(s) unique to syngas and site(s) shared between two conditions; Fructose / Syngas & Fructose, site(s) unique to fructose and site(s) shared between two conditions; Syngas, site(s) unique to syngas; Fructose, site(s) unique to fructose; Fructose / Syngas & Fructose, site(s) unique to fructose and site(s) shared between two conditions; Fructose / Syngas / Syngas & Fructose, site(s) unique to syngas, site(s) unique to fructose, and site(s) shared between two conditions; Fructose / Syngas, site(s) unique to syngas and site(s) unique to syngas. Bubbles icon designed by kerismaker from Flaticon (www.flaticon.com). Sugar-cube icon designed by Freepik from Flaticon (www.flaticon.com).

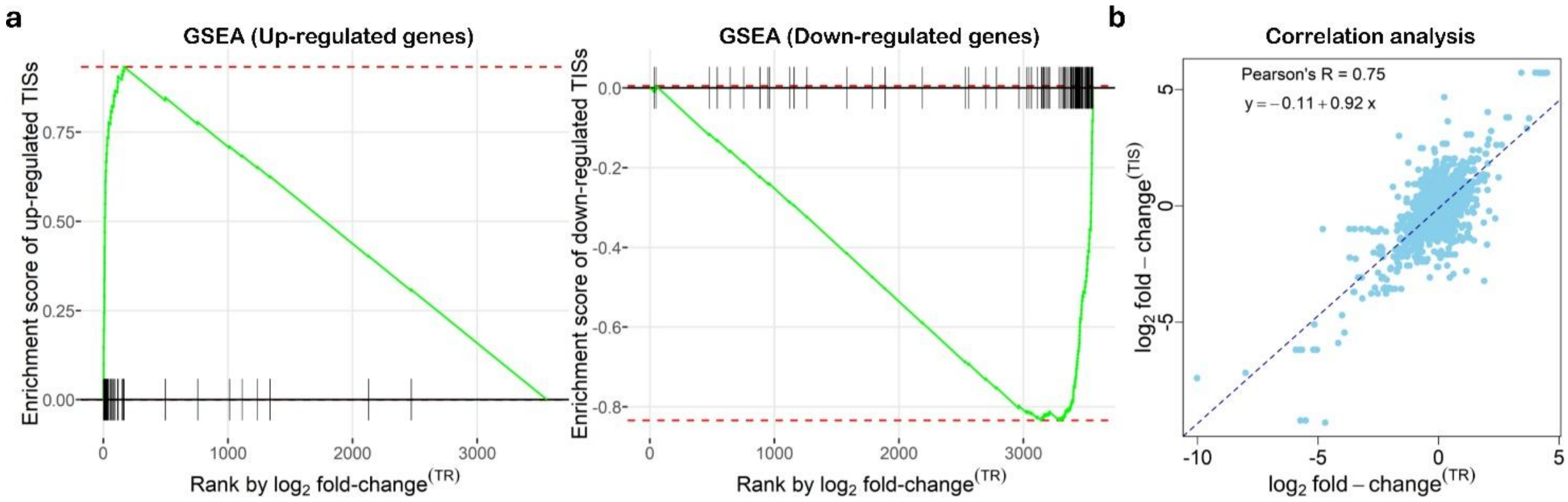

Supplementary Fig. 9. Relationship between differential transcription initiation signals and transcript expression between syngas and fructose *C. autoethanogenum* chemostats. a Enrichment of genes with differential transcription initiation signals within top or bottom ranks of transcript expression changes according to gene set enrichment analysis (GSEA) using the fgsea package in the software R at FDR 5%. Black lines indicate the location of genes with differential signals of transcription initiation while red lines indicate the upper and lower bounds of the enrichment score. TIS, transcription initiation site. **b** Correlation and quantitative relationship between $\log_2$ fold-changes of TIS signals and respective transcript expression. Correlation is quantified by Pearson's correlation coefficient (R) and magnitude of effect by the slope of the linear regression fit.

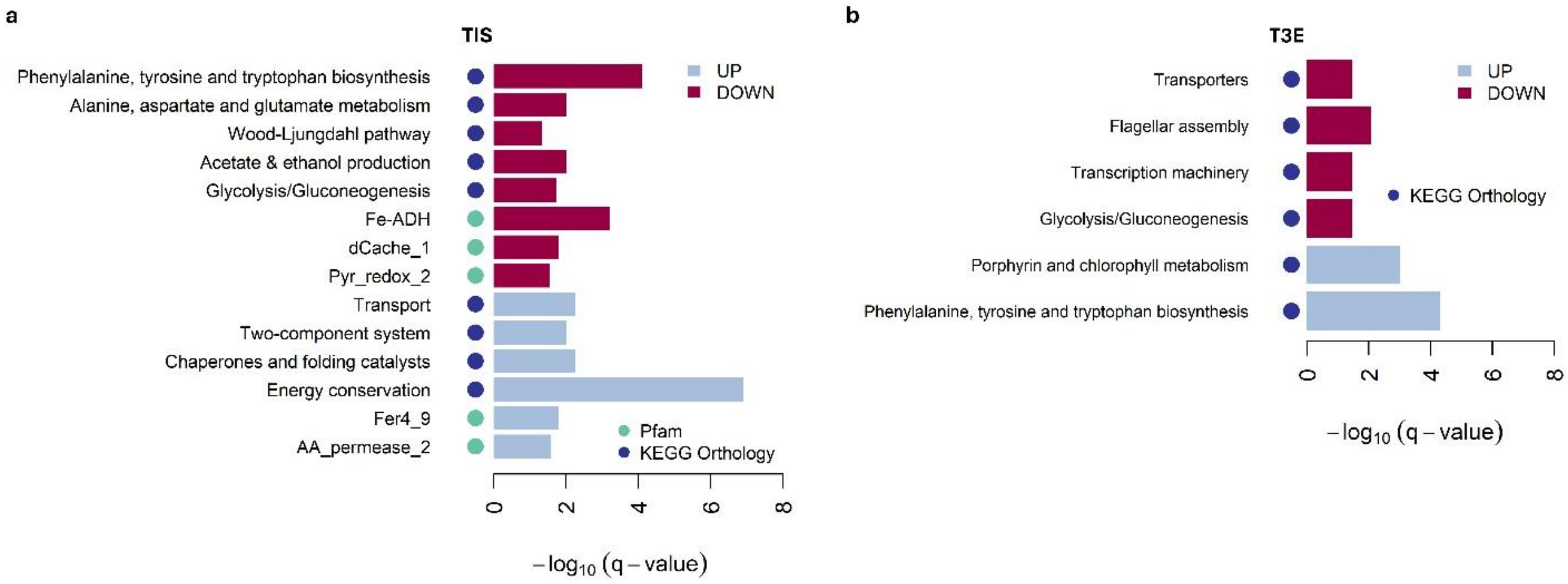

Supplementary Fig. 10. **Functional enrichment of genes featuring differentially quantified TISs and T3Es**. **a** The barplot shows the functional classes enriched in genes featuring differentially quantified TISs by hypergeometric test (FDR = 5%). Pfam, Pfam entries (DOI: 10.1093/nar/gkaa913) from eggNOG-mapper 2.1.12 (DOI: 10.1093/molbev/msab293); KEGG Orthology, KEGG Orthology (KO) functional categories (DOI: 10.1016/j.jmb.2015.11.006) from Valgepea *et al.* (Level 3 in Table S3; DOI: 10.1128/msystems.00026-22). **b** The barplot shows the functional classes enriched in genes featuring differentially quantified TISs by hypergeometric test (FDR = 5%).

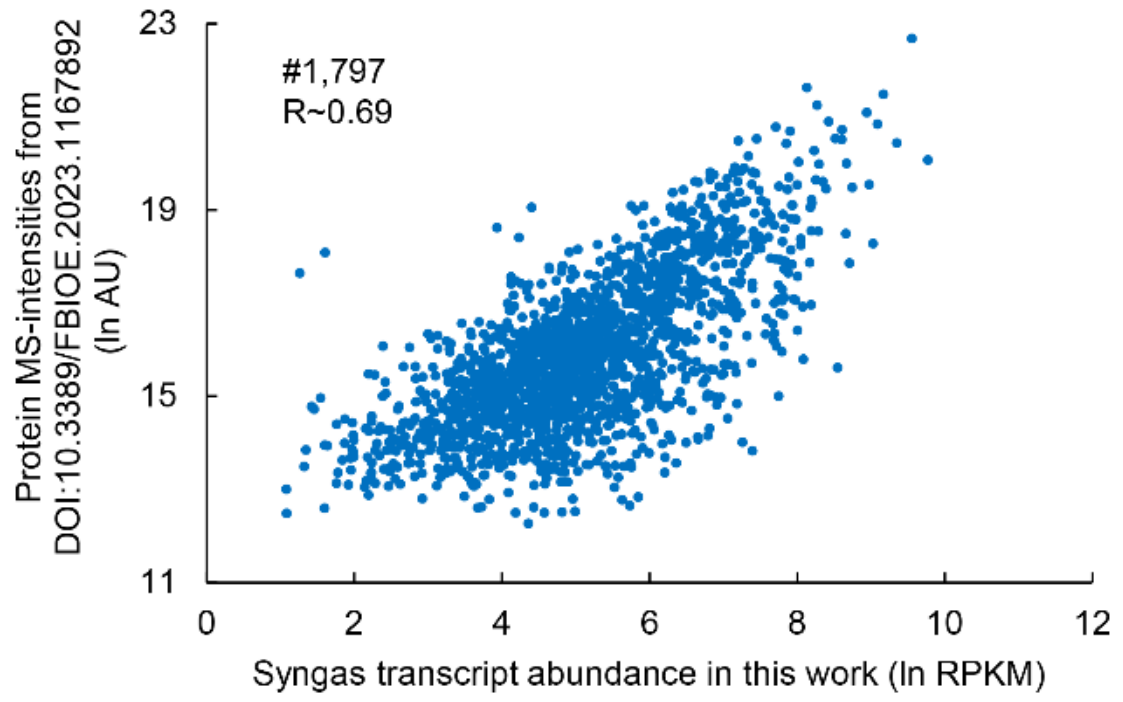

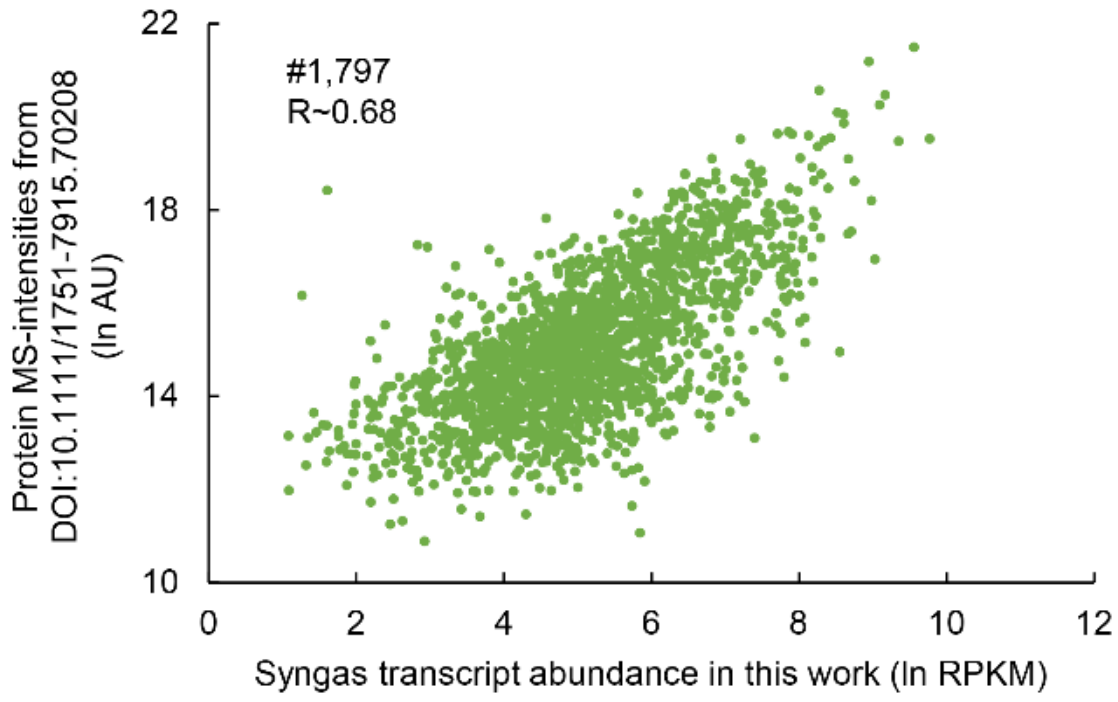

Supplementary Fig. 11. **Correlation between transcript levels quantified in this work for *C. autoethanogenum* syngas chemostats and protein levels quantified in previous works for *C. autoethanogenum* syngas chemostats.** All datasets from *C. autoethanogenum* strain LAbrini chemostats operated at pH 5, 37°C, and dilution rate 1 day^{-1}. R, Pearson product correlation; RPKM, reads per kilobase of transcript per million mapped reads; MS, mass spectrometry.
