# Supplementary material for: Systems-level analysis predicts no autotrophy-linked protein-RNA interactions in Clostridium autoethanogenum
Source: Nat Commun. 2026 Jul 8;17:8435. doi: 10.1038/s41467-026-75309-w (PMC13478517; doi:10.1038/s41467-026-75309-w)
Supplement: Supplementary file 2 [file 41467_2026_75309_MOESM2_ESM.pdf]

nature portfolio

Peer Review File

Systems-level analysis predicts no autotrophy-linked protein-RNA interactions in Clostridium autoethanogenum

Corresponding Author: Professor Angela Re

This file contains all reviewer reports in order by version, followed by all author rebuttals in order by version.

Version 0:

Reviewer comments:

Reviewer #1

(Remarks to the Author)
The manuscript by Re and coworkers deals with elucidating protein-RNA interactions as a regulatory layer of acetogen metabolism. As such, it addresses a hitherto neglected part of how autotrophy in acetogens could be regulated. The study and its data are very high quality and the findings significantly push forward our understanding of how this ancient type of metabolism functions. I congratulate the authors to this achievement.
Prior to dissemination to the public, I recommend to address a couple I have listed below mainly related to improving clarity of the manuscript. My main point in this respect is that the way the different types of regulation are addressed should be unified (“transcriptional”, “translational”, “posttranscriptional” and “posttranslational” regulation) throughout the manuscript. Additionally, since the main finding was that RBP-level regulation does not appear to be the dominant mode of regulation in C. autoethanogenum, and potentially other/all acetogens, it would be important to communicate this result as such without implying a role in autotrophy. This is the main finding of this study, which is significant and impactful. To embed this result in a bigger context, I suggest to use part of the discussion to paint the big picture of the different layers of regulation in acetogens. The authors have already done this by contextualizing their results (lines 412-433) but this point should be stressed even more, potentially also supported by a figure summarizing the different layers of regulation.

Points suggested to be addressed:

Abstract:
• Remove “for the first time” from abstract
• Remove statement about involvement of RBP-level regulation of autotrophy

Introduction
• Line 53: remove “for the first time”
• Line 54-56: this sentence has a rather complicated structure. Split into two sentences?
• Line 82-83: please explain what catRAPID does for “multi-parametric rating in an organism-agnostic framework“

Results
• Line 109: clarify that “both” refers to fructose and syngas for LAbrini
• Line 114-115: remove sentence stating that polysome profiling has only been used for few microbes from text or explain why this is important information
• Line 121-122: explain in the text based on which criterion the outliner was identified and/or excluded from further analysis
• Line 132-136: it is not entirely clear from the description how the comparison was done. How were the calculated ratios of the ribosome-bound/ribosome-free fraction for each substrate compared?
• Line 139-143: explain clearer which information is gained from the second and third metric
• Line 147-148: it is not clear to which base number the 29% unique genes are referring
• Line 150-154: this sentence is not clear and should be streamlined to make clear what the CONSENSUS approach does
• Line 177-178: state the full locus tag of NuoF and to which enzyme complex it belongs
• Line 181: Energy conservation is not a single pathway. I suggest to rephrase this sentence and to remove “&” from the sentence
• Line 183-195: give full locus tags for each gene
• Line 187: insert “the” after “generates”

• Line 193-195: explain why buffering effects might be the cause for discordant changes in transcriptional/translational regulation
• Line 210: insert “sites” after “internal”
• Line 213: write “2221” as “2,221”
• Line 233-234: state why not TISs for the remaining protein-coding genes were identified
• Line 252: exchange “on” with “in”
• Line 282-285: remove color references from the text
• Line 294: Does “ID 04855” refer to a locus tag? If yes, please state the full locus tag
• Line 294-295: explain GAF and PAS
• Line 296: explain REC
• Line 310-311: what are the implications of this observation?
• Line 328-330: above (line 287-289), it was stated that syngas-specific TISs were identified for genes of the Rnf complex in addition to TISs share between syngas and fructose. Could a comment be added to the text reflecting on how the specific TISs might have to the higher expression levels of Rnf genes in addition to stronger signals for the shared TISs.
• Line 334: replace “with” with “, including”
• Line 336-339: clarity of the sentence should be improved
• Line 352: add explanation how “to encode sequence-derived biochemical and structural features” is done and how many potential RBPs were identified
• Line 363-368: clarity of this paragraph needs to be improved. The message is not clear. E.g., what does “genes involved in … were mediated by RBPs …” mean?
• Line 370: the number of 69 target genes showed be clearly named in the paragraph before to make clear from where this number comes from.
• Line 387: change “by-products” to “products”

Discussion
• Should be streamlined in my opinion. It is now packed with information and discussion points. Reducing the information density together with increasing clarity in the language and sentence construction, would in my opinion be beneficial to convey the central messages of this study
• Line 410-411: this sentence suggests the possibility of widespread translational regulation, which the following sentences are arguing against. Suggest to rephrase this. The arguments presented from line 412-433 are in my opinion crucial for describing the regulatory network of acetogens (as far as it is known now) and should be expanded (see my comment above)
• Line 467: Change “the most” to “the highest number of”
• Line 501-502: would tune that statement down since no interaction was determined which is directly linked with autotrophy. For this reviewer, this is the significant achievement of this study, elucidating that this type of regulation does not appear to play a major role in regulating acetogen metabolism.

Methods
• Cultivation conditions: report gassing rates + agitation for syngas and heterotrophic conditions together in one sentence, and remove second reference to fructose concentration in the paragraph
• Line 519-520: write “g/L biomass or cell dry weight” instead of “gram of dry cell weight per litre”

Figures

Figure 2:
• describe cut-off for DTR in figure legend
• the inset of figure 2b lacks a y-axis scale. Please insert.
Figure 3:
• Color of PRONTO-TK in annotation source does not match color of “candidate RBPs” in Figure 3b

Figure 6:
• What is the central circle in the figure representing?
• The text legend should explain in more detail the symbol legend shown in the figure
• SORD should be explained in the figure legend
• CoFeSP and THF should be explained in the legend

(Remarks on code availability)

Reviewer #2

(Remarks to the Author)
The manuscript by Re et al presents a multi-omics study that combines chemostat cultivation, RNA-seq, and computational tools to investigate gene controls in the model acetogen Clostridium autoethanogenum. In addition to regular transcriptomic RNA-seq, the authors have conducted multiple variants of RNA-seq, including ribo-seq (polysome-seq) to analyze translating mRNAs, Cappble RNA-seq to identify transcription start sites, and Term-seq to identify transcription termination sites. This analysis generated global RNA maps of transcription start and termination sites, which could be a fine resource for RNA research in C. autoethanogenum. However, comparing the differential gene expression profile at two different

growth conditions (syngas vs. fructose), the authors identified little changes that are of significance in the acetogen biology. The authors further attempted to predict potential RNA-binding proteins (RBP) in C. autoethanogenum, but failed to confirm any of the predicted RBP-RNA interactions and to demonstrate their contribution to autotrophy.
Specific comments are below:

- Line 50. No RBPs have been described in acetogens so far, but several known RNA-binding proteins have been characterized in its close-related relatives Clostridium diffcile, including various ribonucleases and Hfq (Fuchs et al, PNAS 2021, PMID: 34131082).
- Line 53, the aim of the work is to identify for the first time in acetogens RBP-RNA interactions. This is unfortunately not achievable by the experimental design, and not achieved by the data collected so far.
- Line 129-143, The quality of the polysome-seq is unclear. Reads coverage are not shown for mRNAs to confirm CDS-only coverage by ribosomes, and for noncoding RNAs that are expected to be little bound to polysomes. It is also unclear whether these polysome-seq data are sufficiently quantitative for protein expression analysis.
- Line 124-128, only the top 5% genes with the lowest q-value was considered as differential expression. Was fold-change even considered? Such strong suppression is unusual and introduces unwarranted bias and conclusions in data analysis, such as the coupling of transcriptional and translation control. The authors' view may be largely distorted from real world gene expression.
- Figure 2, Limited transcriptional and translation control. Is the control really limited or suppressed in data analysis?
- Line 173 and Figure 3, Uncoupled transcriptional and translational control. This is overstatement and inaccurate. The exact concept of coupling is not defined in the manuscript, and lacks well-defined controls.
- Line 155-156, translation genes are strongly enriched in DTLs. Why is this reasonable and expected?
- Although the authors have mapped thousands of TIS and T3S, few are different between syngas and fructose conditions. nothing new has been learned by doing such analysis.
- Line 346, this entire section of RBP analysis is highly speculative. The predicted candidate RBPs are mostly known enzymes in RNA metabolism. PRONTO-TK is a prediction software based on function annotations, which lacks the power to identify new RNA-binding proteins. None of the predicted RBP or RBP-RNA interactions has been experimentally verified, adding little to the relevant field.
- Most figures are too abstract, showing little results and real data.

(Remarks on code availability)

Reviewer #3

(Remarks to the Author)
Re et al. present a manuscript in which they collect multi-omics data on translation and RNA expression in the model acetogen Clostridium autoethanogenum, and then apply sophisticated computational analyses to identify the major drivers of gene-expression changes between autotrophic and heterotrophic growth conditions. They show that transcriptionally regulated genes and translationally regulated genes overlap only minimally, suggesting a decoupling of translational control from RNA-level control. In addition, they map 5′ and 3′ transcript ends and examine condition-dependent changes in end-site usage. Finally, they use one computational tool to predict RNA-binding proteins (RBPs) and a second tool to predict RBP binding to differentially regulated genes.
Overall, I think this work will be a valuable resource for the bacterial community, especially researchers interested in acetogens. It should also be of interest to the broader gene-expression regulation community, as it provides a useful blueprint for integrating multiple gene-expression datasets and applying emerging tools to infer potential regulatory circuits. I consider the manuscript a strong candidate for publication, but I do think a few issues should be addressed prior to acceptance.

Major points
1. Limited overlap between methods in the DTL analysis.
In the DTL analysis, the authors compare three approaches for identifying translationally regulated genes. They note that the overlap of regulated genes across methods is minimal and argue that this reflects important differences between the analyses. I would encourage a more cautious interpretation. Differential regulation depends on thresholds that are necessarily somewhat arbitrary, and many genes may fall just above or below a cutoff depending on the method. Rather than focusing only on the subset that passes a hard threshold, it would be informative to compare the distributions of scores/effect sizes across methods.
For example, for genes called differentially regulated by TL-status, where do they fall in the percentile distribution when genes are ranked by TL-profile? Is there a significant shift relative to random expectation? If there is, then the methods may be capturing similar trends even if they disagree on which genes cross the cutoff.
2. A similar analysis as just mentioned above would also help clarify the apparent overlap (or as pointed out by the authors the current lack thereof) between DTR and DTL gene sets.
3. Regulatory network predictions are discussed too definitively.
A major concern with the Discussion is that the inferred regulatory networks are sometimes described in a way that reads as if the interactions are established. However, these networks are predictions produced by multiple computational steps (e.g., an algorithm to predict RBPs and another to predict binding sites/targets). The authors do not present experimental validation demonstrating that these interactions occur in vivo. The Discussion should more clearly reflect the predictive nature of these results and more explicitly discuss limitations, sources of uncertainty, and potential false positives/negatives in the computational pipeline.

I am not suggesting that experimental validation is required for publication, but a more balanced discussion - particularly one that more transparently frames these networks as hypotheses - would help readers interpret the predictions appropriately.
4. Directionality of DTL changes (all upregulated in syngas).
For the DTL genes, why are all (or nearly all) upregulated in syngas relative to fructose? This pattern raises the possibility of a normalization or scaling issue, or alternatively a global shift in translation capacity/efficiency between conditions. The authors should address this explicitly and discuss potential technical and biological explanations.
5. Clarify the TIS/T3E category definitions in Figure 5e.
In Figure 5e, the categorization of TIS and T3E sites is not immediately clear. The “Syngas & Fructose” category (shared sites) is understandable, but categories such as “Syngas / Syngas & Fructose” are ambiguous based on the current description. My interpretation is that these refer to genes with one site shared between conditions and an additional site specific to syngas; however, this should be stated explicitly. Similarly, does “Fructose / Syngas” refer to genes that have a condition-specific site in each condition but no shared site?
A supplemental schematic illustrating each category with simple gene models would substantially improve clarity.

Minor points
5. Lines 336–339: What is the quantitative relationship between changes in transcription-initiation signal strength and differential transcript expression? Are they simply directionally consistent, or is the magnitude of the effect correlated?
6. Interpretation of DTR vs DTL gene sets: This is a subjective interpretation from my side, but the data could be read as though DTR genes are more condition-specific, whereas DTL genes appear more “housekeeping”-like. This aligns with a general regulatory “big picture,” where transcription can act like a faucet for turning condition-specific genes on/off, while translation fine-tunes protein output for genes expressed across many conditions. Do the authors agree with this framing? If so, it might be worth a brief mention (with appropriate caveats).
7. Figure 3A: The Venn diagrams are visually busy. An UpSet plot might communicate the overlaps more clearly. Alternatively, removing the “DTL (down)” group (it has no entries anyway) would reduce clutter.
8. Figure 4: In the heuristic procedure panel (second row with four rank plots), the leftmost plot lacks a title. If it is intended to be “Rank by coverage,” please add a title consistent with the other three panels.
9. Readability / take-home messages: At points, the text becomes difficult to follow due to dense, multi-step descriptions. For example, the paragraph spanning lines 287-303 discusses multiple regulated and connected biological processes, but the key take-home message is not clear. Consider adding occasional simplifications and explicit summary sentences.
10. Introduction length/detail: The Introduction currently includes substantial procedural detail about what was done. This could likely be shortened; many of those details fit better in Results/Methods.
11. Citations: Please check citation choices and accuracy (e.g., the RIC citations around line 57). Where appropriate, consider citing original primary literature in addition to a review.
12. Lines 56–60: Strictly speaking, methods such as XRNAX or OOPS (not poly(A)-dependent) could also be used to identify RBPs. The current sentence reads slightly as if it is justifying the chosen approach in a way that is not fully aligned with the broader methodological landscape. The text could be tweaked for precision while keeping the intent.
13. Line 97: Consider briefly defining autotrophic vs heterotrophic in parentheses. While obvious to many readers, this will likely interest a broader gene regulation audience where basic terms may benefit from quick clarification.
14. Line 391 (terminology): Minor wording point: “protein translation” is imprecise as it technically does not exist; consider “mRNA translation” or “protein synthesis/production.”

(Remarks on code availability)

Version 1:

Reviewer comments:

Reviewer #1

(Remarks to the Author)
All points I previously raised have been suitably addressed by the authors. I would like to congratulate them again to their work.

(Remarks on code availability)

Reviewer #2

(Remarks to the Author)
In the revised manuscript, the authors have clarified the limitations of bioinformatic predictions and provided a more balanced view/discussion. While this reviewer does not find any of the findings/ conclusions particularly exciting, the presented data may serve as a useful resource for researchers in the field.

(Remarks on code availability)

Reviewer #3

(Remarks to the Author)
The authors have addressed nearly all of the points I raised in my initial review, and I support publication. I have only one small clarification that I would find useful if the authors could address it.

I previously asked for an analysis of the overlap, or lack thereof, between DTR and DTL genes. The authors performed this analysis and wrote that they conducted "such analysis by ranking genes according to their q-values in differential transcription analysis and according to their ranks from the CONSENSUS approach in differential translation analysis. Analysis of gene counts across bins of ranks (Supplementary Fig. 6 below) showed weak agreement in highly ranking genes between the two analyses…". However, looking at Supplementary Figure 6, I do not see even weak agreement. I would appreciate it if the authors could provide a more detailed explanation of why they interpret these data as showing weak agreement.

Aside from this minor point, I have no other concerns about the current manuscript. There are a few minor typos and grammatical issues throughout the manuscript. For example, in the Supplemental Methods text for Approach A, the authors write: "We carried out his procedure for each pair of the three metrics." I assume "his" should be "this." These issues should be easily addressed by a thorough editorial pass over the text.

(Remarks on code availability)

Open Access This Peer Review File is licensed under a Creative Commons Attribution 4.0 International License, which permits use, sharing, adaptation, distribution and reproduction in any medium or format, as long as you give appropriate credit to the original author(s) and the source, provide a link to the Creative Commons license, and indicate if changes were made.
In cases where reviewers are anonymous, credit should be given to 'Anonymous Referee' and the source.
The images or other third party material in this Peer Review File are included in the article's Creative Commons license, unless indicated otherwise in a credit line to the material. If material is not included in the article's Creative Commons license and your intended use is not permitted by statutory regulation or exceeds the permitted use, you will need to obtain permission directly from the copyright holder.
To view a copy of this license, visit https://creativecommons.org/licenses/by/4.0/

Responses to reviewer comments

Dear reviewers,

We want to thank you for reviewing our manuscript "Systems-level analysis identifies protein-RNA interactions contributing to autotrophy of *Clostridium autoethanogenum*". We appreciate that the reviewers acknowledge the relevance of our work for both the more specific acetogen community and the wider gene-expression community. However, the reviewers requested modifications to enhance the quality of the manuscript, which we hope to have thoroughly addressed. We include a copy of the revised manuscript named "Manuscript revised_with track changes" and in our responses we refer to line numbers in that file with the Simple Markup view in Word. The revised manuscript includes minor changes we made in addition to the changes addressing reviewer's comments (e.g. typos, reference formatting, inconsistent numbering). We also corrected the text on the predicted RBPs in lines 393-400 and Fig. 7 due to amistake in handling the predicted RBP dataset for our initial submission. In the current response file, reviewer comments are written in black and answers to comments in blue font.

Reviewer #1 (Remarks to the Author)

The manuscript by Re and coworkers deals with elucidating protein-RNA interactions as a regulatory layer of acetogen metabolism. As such, it addresses a hitherto neglected part of how autotrophy in acetogens could be regulated. The study and its data are very high quality and the findings significantly push forward our understanding of how this ancient type of metabolism functions. I congratulate the authors to this achievement.

Prior to dissemination to the public, I recommend to address a couple I have listed below mainly related to improving clarity of the manuscript. My main point in this respect is that the way the different types of regulation are addressed should be unified ("transcriptional", "translational", "posttranscriptional" and "posttranslational" regulation) throughout the manuscript. Additionally, since the main finding was that RBP-level regulation does not appear to be the dominant mode of regulation in C. autoethanogenum, and potentially other/all acetogens, it would be important to communicate this result as such without implying a role in autotrophy. This is the main finding of this study, which is significant and impactful. To embed this result in a bigger context, I suggest to use part of the discussion to paint the big picture of the different layers of regulation in acetogens. The authors have already done this by contextualizing their results (lines 412-433) but this point should be stressed even more, potentially also supported by a figure summarizing the different layers of regulation.

Response: We thank the reviewer for acknowledging the quality and relevance of our work. We have addressed all the valuable comments raised by the reviewer, as detailed below, which has considerably improved the quality of the manuscript in our opinion. Regarding the potential additional figure summarizing different layers of regulation, we feel that maintaining two 'abstract-type' figures (Fig. 1 and 4) is potentially preferable considering the comment from Reviewer #2 on figures.

Points suggested to be addressed:

Abstract:
• Remove “for the first time” from abstract

Response: We have removed the term and revised wording of the sentence based on the next comment as follows:

L18-21: In this work, we aimed to identify genome-wide protein-RNA interactions contributing to autotrophy in the model-acetogen *Clostridium autoethanogenum* by combining steady-state chemostat cultivation, functional genomics, and computational methods.

• Remove statement about involvement of RBP-level regulation of autotrophy

Response: We thank the reviewer for pointing out that our wording was misleading and that we should clearly communicate the result of not predicting autotrophy-specific RBPs, as stated in the introductory comment by the reviewer. We thus revised the wording of this particular sentence as specified in response to the previous comment. Additionally, we revised the title of the manuscript and wording of sentences throughout the manuscript that were potentially implying RBP contributions to autotrophy as follows:

Title (L1-2): Systems-level analysis predicts no autotrophy-linked protein-RNA interactions in *Clostridium autoethanogenum*

L24-26: We then used computational tools to reconstruct protein-RNA interactions for differentially regulated genes, predicting 14 trans-acting regulatory RNA-binding proteins (RBPs) involved in post-transcriptional regulation but not linked to genes key for autotrophy.

L63-64: Fig. 1. Integrative functional genomics and computational approach used in this work to predict RNA-binding proteins (RBPs) and protein-RNA interactions in *C. autoethanogenum*.

L70-71: Last, we used the neural network-based toolkit PRONTO-TK4[5] to predict potential RBPs and the inferential tool catRAPID[46] to computationally reconstruct RBP-RNA interactions in *C. autoethanogenum*.

L84-86: To map genome-wide RBP-RNA interactions that could contribute to autotrophy in acetogens, we grew the *C. autoethanogenum* strain LAbrini[36] on syngas (CO, H_2, CO_2) and fructose in chemostat continuous cultures (Fig. 1).

L415: Fig. 7. RNA-binding proteins (RBPs) linked to differentially regulated genes in *C. autoethanogenum*.

L426-428: To address these gaps with this work, we employed a comprehensive approach by combining chemostat cultures, functional genomics, and computational methods to predict genome-wide RBPs and RBP-RNA interactions in the model-acetogen *C. autoethanogenum*.

L538-539: Secondly, we report predicted genome-wide protein-RNA interactions for differentially regulated genes in a model-acetogen.

Introduction

• Line 53: remove “for the first time”

Response: We have removed the term and revised the sentence as follows:

L53-54: The aim of our work was to identify genome-wide RPB-RNA interactions that contribute to autotrophy in acetogens.

We also removed the same term from a sentence in the last paragraph of Discussion, which now reads:

L538-539: Secondly, we report predicted genome-wide protein-RNA interactions for differentially regulated genes in a model-acetogen.

• Line 54-56: this sentence has a rather complicated structure. Split into two sentences?

Response: We thank the reviewer for this suggestion and have thus split the respective sentence into two sentences:

L54-56: We used the model-acetogen *Clostridium autoethanogenum* as its metabolism has been explored through numerous omics datasets[3,33-38]. In addition, it has been engineered to produce various non-native chemicals[3,39,40] and it is used in commercial-scale gas fermentation for ethanol production[2].

• Line 82-83: please explain what catRAPID does for “multi-parametric rating in an organism-agnostic framework“

Response: We thank the reviewer for pointing out the need for better clarity here. catRAPID uses a multi-factorial rating system to rank the predicted protein-RNA interactions. The factors include the interaction propensity, the RBP propensity, and presence of known RNA-binding motifs. The interaction propensity scores the interaction ability between one protein (or region) and one RNA (or region). This score is based on the observed tendency of the components of ribonucleoprotein complexes to exhibit specific properties of their physical-chemical profiles (DOI: 10.1038/nmeth.1611). The RBP propensity is a measure of the propensity of the protein to bind RNA based on its similarity to a RBP precompiled library. This catRAPID approach can be defined as organism-agnostic since it can accept custom protein and custom RNA sequences and therefore can be applied to any organism.

Considering a comment from Reviewer #3 (see comment ‘10. Introduction length/detail:…’) on excessive procedural details in our Introduction section, we shortened that paragraph considerably and this also included removal of ‘multi-parametric rating in an organism-agnostic framework’ on catRAPID. We thus addressed this comment by adding details on the rating system of catRAPID in the Methods section as follows:

L781-785: We employed catRAPID omics v2[46] to predict protein–RNA interactions between the candidate RBPs and differentially regulated genes (DTRs and DTLs) at genome-scale. The tool accepts custom input protein and RNA sequences and computes interaction scores based on updated precompiled libraries. The multi-parametric rating system of catRAPID predictions integrates multiple scores[141] – interaction propensity, RBP propensity, and presence of known RNA-binding motifs – to provide final scores ranging between 0 and 1.

Results

• Line 109: clarify that "both" refers to fructose and syngas for LAbrini

Response: We thank the reviewer for highlighting the need for more clarity here and have thus modified the sentence as follows:

L98-101: Interestingly, both syngas and fructose chemostat cultures with the LAbrini strain showed only ~1.5-fold higher acetate production than ethanol (Supplementary Fig. 1a) while nearly no ethanol production was detected in fructose-grown batch cultures of *C. autoethanogenum* strain JA1-1[50].

• Line 114-115: remove sentence stating that polysome profiling has only been used for few microbes from text or explain why this is important information

Response: We have removed the sentence.

• Line 121-122: explain in the text based on which criterion the outliner was identified and/or excluded from further analysis.

Response: We thank the reviewer for highlighting the insufficient level of detail here. We modified the sentence to include the criteria while keeping the description of the detailed procedure in the Methods section (L708-726).

L111-113: Combined correlation analysis, compositional distance analysis, and principal component analysis of polysome-seq data (see Methods for details) led to the removal of an outlier bio-replicate culture for both syngas and fructose (Supplementary Fig. 4).

• Line 132-136: it is not entirely clear from the description how the comparison was done. How were the calculated ratios of the ribosome-bound/ribosome-free fraction for each substrate compared?

Response: We thank the reviewer for pointing out the need for more clarity here. We thus split these lines into more sentences and modified text as follows:

L123-128: First, we calculated the ratio between the sum of the mRNA proportions quantified in the ribosome-bound and ribosome-free fractions per gene for both substrates. This is equivalent to the term 'ribosome occupancy' traditionally used for polysome-seq data[51]. We then quantified the difference in this translation status metric between syngas and fructose (TL-STATUS) by subtracting the fructose ratio from the syngas one. Positive value for TL-STATUS thus indicates up-regulation and negative down-regulation on syngas vs fructose (Supplementary Table 4).

Accordingly, we also modified the legend text for Fig. 2b TL-STATUS inset as follows:

L161-163: For the TL-STATUS metric, inset shows the calculated translation status (*i.e.* ratio between the sum of the mRNA proportions quantified in the ribosome-bound and ribosome-free fractions).

• Line 139-143: explain clearer which information is gained from the second and third metric

Response: We thank the reviewer for highlighting the need for more explanation here. As explained more thoroughly in our response to the previous comment, the first metric – TL-STATUS – quantifies the difference in the translation status (*i.e.* ratio between the sum of the mRNA proportions quantified in the ribosome-bound and ribosome-free fractions per gene for both substrates). Thus TL-STATUS captures

the overall translation status of all mRNA for a gene while the second – TL-PROFILE – and third – POLY-FRACTIONS – metrics provide different information by considering the individual polysome fractions to identify consistent changes between growth conditions. Specifically, TL-PROFILE considers the profile of individual fractions down-weighting fractions with higher variability and quantifies the distance of the profiles between the two conditions. POLY-FRACTIONS, however, identifies individual fractions that consistently change between growth conditions by relying on a bootstrapping procedure and prioritizes genes according to the number of statistically significant different fractions. We thus added the following sentence to the respective paragraph to clarify these aspects:

L134-137: While TL-STATUS captures the overall translation status of mRNAs for any gene, POLY-FRACTIONS and TL-PROFILE provide additional information on translational regulation by capturing differences between individual polysome fractions and their profiles, respectively.

• Line 147-148: it is not clear to which base number the 29% unique genes are referring

Response: We thank the reviewer for pointing out this shortcoming. The 29% refers to the average % of the unique genes each metric captures (Fig. 2c left part) - 21.3, 32.2, 32.7. We have specified this in the revised sentence as follows:

L141-143: Interestingly, each metric identified ~29% (21.3, 32.2, 32.7%) unique genes and also unique KEGG Orthology (KO) functional categories[55] or Pfam entries[56] were enriched within the top 5% gene sets of each metric (Fig. 2d).

• Line 150-154: this sentence is not clear and should be streamlined to make clear what the CONSENSUS approach does

Response: We thank the reviewer for highlighting the need for a better explanation here. We thus rephrased the sentence as follows:

L145-147: Therefore, we decided to determine DTLs using a CONSENSUS approach: firstly, we calculated for each gene its average rank across the three metrics; and secondly, we ranked these average ranks and defined DTLs as the genes in the top 5%.

• Line 177-178: state the full locus tag of NuoF and to which enzyme complex it belongs

Response: We thank the reviewer for this comment.

Regarding writing out the full locus tag here, we believe that if we do that, we should use the full tag (i.e. with 'LABRINI_') for all gene IDs in the manuscript. We feel that this could compromise the readability of the manuscript as text would become longer and scattered with repeats of 'LABRINI_'. We would thus prefer to maintain our approach of writing out the full tag at first use (*i.e.* for the first gene mentioned in the text). To make this clearer, we have modified the respective part of the sentence as seen below.

Regarding the enzyme complex for NuoF, we have added the information as seen below:

L173-176: While no DTLs were down-regulated on syngas vs fructose, the concordantly up-regulated six genes included the Fd-NAD+ oxidoreductase Rnf complex subunit RsxC (LABRINI_16130; following gene IDs stated without 'LABRINI_') and the NADH-quinone oxidoreductase complex subunit NuoF (07815) (Supplementary Tables 3 and 4).

• Line 181: Energy conservation is not a single pathway. I suggest to rephrase this sentence and to remove "&" from the sentence

Response: We thank the reviewer for pointing out this shortcoming in our text. We agree that energy conservation is not a pathway. We aimed to refer to specific KEGG Orthology (KO) functional categories here. We have thus modified the sentence as follows:

L184-186: Furthermore, the enrichment data show that two key metabolic modules for autotrophy – KO functional categories 'Acetate & ethanol production' and 'Energy conservation' – are only regulated at transcript levels while ribosomal proteins are only regulated at translational level (Fig. 3b).

We addressed the same shortcoming also later in Results by modifying the sentence:

L321-323: For T3Es, the KO functional category 'Acetate & ethanol production' was enriched in genes displaying TE3s specific to either syngas or fructose (Fig. 5f), indicating the potential relevance of regulation of termination in expression of genes involved in product synthesis.

We note that we now abbreviate KEGG Orthology as 'KO' at first use in text as seen below. In the original manuscript, this abbreviation only appeared in relevant figure legends.

L141-143: Interestingly, each metric identified ~29% (21.3, 32.2, 32.7%) unique genes and also unique KEGG Orthology (KO) functional categories[55] or Pfam entries[56] were enriched within the top 5% gene sets of each metric (Fig. 2d).

• Line 183-195: give full locus tags for each gene

Response: Please see our response above to the comment of 'state the full locus tag of NuoF…'

• Line 187: insert "the" after "generates"

Response: We thank the reviewer for pointing out this mistake. We have made the respective change.

• Line 193-195: explain why buffering effects might be the cause for discordant changes in transcriptional/translational regulation

Response: We thank the reviewer for highlighting the need for more clarity here. In the interest of readability, we removed the 'buffering point' from this sentence and added a new sentence on this as follows:

L197-201: Only four genes showed discordant changes between transcriptional and translational regulation, with LABRINI_07435 and LABRINI_00130 putatively involved in iron metabolism[65,66] (Supplementary Tables 3 and 4). Discordant changes could potentially result from condition-specific regulation of translational efficiency, particularly to maintain stable iron metabolism, as the iron composition of the medium remains unchanged between the two conditions.

• Line 210: insert "sites" after "internal"

Response: We thank the reviewer for this comment and we have made the respective change.

• Line 213: write "2221" as "2,221"

Response: We thank the reviewer for pointing out this mistake. We have made the respective change.

• Line 233-234: state why not TISs for the remaining protein-coding genes were identified

Response: We thank the reviewer for this comment. We would prefer to avoid adding any statements about the reasons for not detecting TISs for more protein-coding genes (>40%) as these would be highly speculative in our view. Firstly, the maximum percentage would not be 100 as many genes are organized in polycistronic operons and would thus not be expected to contain TISs for all operon genes. We currently lack experimental data for *C. autoethanogenum* quantifying actual transcriptional units, thus we do not know the 'maximal percentage' that could be achieved with our approach (or for any other approach determining TISs). Secondly, even established next-generation sequencing platforms, which were used in this work, have limits for detection and reliable quantification of primary transcripts. Thirdly, the heuristic data analysis procedure we used to identify TISs was prioritized for quality (*i.e.* reliability), not for quantity. Altogether, we see that it would be very speculative to try to estimate the contribution of these potential effects on the TIS coverage for protein-coding genes.

• Line 252: exchange "on" with "in"

Response: We thank the reviewer for pointing out this mistake. We have made the respective change.

• Line 282-285: remove color references from the text

Response: We feel keeping the color references is beneficial for the reader to more easily and quickly link the text statements to the figure without needing to read the full legend text (which is extended in the revised manuscript based on a valuable comment from Reviewer #3). We believe that this comment might have arisen from our mistake of excluding the figure references from within the parentheses in these sentences at first use. We thus modified the sentences as seen below to improve clarity:

L293-298: However, RNA processing at 3'UTRs appears to be more dependent on growth substrates as ~12% of genes show TISs exclusive to fructose (Fig. 5e; light purple) with another ~7% which show exclusive TISs for fructose along with shared TISs (green). Interestingly, a glyceraldehyde-3-phosphate dehydrogenase (08720) with an accompanying transcriptional regulator (08725) showed the most versatile T3E usage with both T3Es exclusive and common for either substrate (Fig. 5e; gray). Notably, we did not detect a single gene showing only T3Es exclusive for syngas.

• Line 294: Does "ID 04855" refer to a locus tag? If yes, please state the full locus tag

Response: We thank the reviewer for highlighting this shortcoming in our text. Since the gene lacks an annotated description, we have modified the sentence to include the full gene ID (locus tag) as follows:

L308-310: Notably, LABRINI_04855 harbors the ligand-binding domains GAF[85] and PAS[86], which can bind carbon monoxide[87], and the HK-A domain, which is the phospho-acceptor domain of HKs.

We added the full gene ID for all genes without annotated descriptions:

L197-201: Only four genes showed discordant changes between transcriptional and translational regulation, with LABRINI_07435 and LABRINI_00130 putatively involved in iron metabolism[65,66] (Supplementary Tables 3 and 4).

L310-312: Furthermore, the two adjacent genes – LABRINI_04850 and LABRINI_04860 – both contain the REC domain[83], which receives the signal from a HK. An additional TCS was enriched here with LABRINI_16335 containing a REC domain and LABRINI_16340 containing the HK-A domain.

• Line 294-295: explain GAF and PAS

Response: We thank the reviewer for pointing out the need for references for these protein domains. We have thus added references into the text as seen below:

L308-310: Notably, LABRINI_04855 harbors the ligand-binding domains GAF[85] and PAS[86], which can bind carbon monoxide[87], and the HK-A domain, which is the phospho-acceptor domain of HKs.

• Line 296: explain REC

Response: We thank the reviewer for highlighting the need for a reference for this protein domain. We have thus added a reference into the text as seen below:

L310-312: Furthermore, the two adjacent genes – LABRINI_04850 and LABRINI_04860 – both contain the REC domain[83], which receives the signal from a HK. An additional TCS was enriched here with LABRINI_16335 containing a REC domain and LABRINI_16340 containing the HK-A domain.

• Line 310-311: what are the implications of this observation?

Response: We thank the reviewer for this comment. Accordingly, we have expanded the sentence as follows but with caution as it's strictly a hypothesis:

L321-323: For T3Es, the KO functional category 'Acetate & ethanol production' was enriched in genes displaying TE3s specific to either syngas or fructose (Fig. 5f), indicating the potential relevance of regulation of termination in expression of genes involved in by-product synthesis.

• Line 328-330: above (line 287-289), it was stated that syngas-specific TISs were identified for genes of the Rnf complex in addition to TISs share between syngas and fructose. Could a comment be added to the text reflecting on how the specific TISs might have to the higher expression levels of Rnf genes in addition to stronger signals for the shared TISs.

Response: We thank the reviewer for this suggestion and have thus modified the sentence accordingly:

L351-352: Thus, the stronger initiation signals plus syngas-specific TISs (Fig. 5f) most likely lead to the enhanced expression of the Rnf complex CDS transcripts (Fig. 3B; Supplementary Table 3).

• Line 334: replace “with” with “, including”

Response: We see that our original sentence is accurate as the ‘with’ is followed by gene groups enriched in genes with weaker initiation signals. Replacing ‘with’ with ‘, including’ would in our understanding leave the part before the comma incomplete without specifying what was enriched, *i.e.* ‘Interestingly, genes with weaker initiation signals on syngas were enriched, including CooS1 carbon monoxide dehydrogenase subunits (15005-15015), genes involved in ethanol production (02655, 16430, 18700, and 19700), genes belonging to glycolysis/gluconeogenesis, and numerous transcription factors (Fig. 6; Supplementary Fig. 10a).’

• Line 336-339: clarity of the sentence should be improved

Response: We thank the reviewer for highlighting the need for more clarity here. As Reviewer #3 also commented on this sentence (see comment ‘5. Lines 336–339:...’), we modified the sentence to both address Reviewer #3 comment and this comment. Regarding this comment, we split the sentence in two and modified wording as follows:

L344-345: Importantly, changes in transcription-initiation signals are consistent both in directionality and magnitude with differential expression of respective transcripts (Supplementary Fig. 9). Genes with up-regulated or down-regulated TISs were enriched within the top or bottom ranks of genes ordered by transcript expression changes, respectively (see Methods for details; Supplementary Fig. 9a). Changes in TIS signals and transcript expression both correlate and match quantitively well (Supplementary Fig. 9b).

• Line 352: add explanation how “to encode sequence-derived biochemical and structural features” is done and how many potential RBPs were identified

Response: We thank the reviewer for this comment.

Regarding extra details on how PRONTO-TK performs encoding of sequence-derived biochemical and structural features, we believe it would be preferential to shortly note these details in the Methods section as the paragraph containing that sentence is already quite long. We thus added the following sentence in the Methods section part ‘Identification of RNA-binding protein’ and the reference to this in the respective sentence as ‘(see Methods for details):

L774-777: The ProtT5-XL model converts protein sequences into 1024-dimensional vector embeddings that capture learned biochemical properties (hydrophobicity, charge, size) and structural propensities (secondary structure preferences, fold patterns) derived from evolutionary relationships in the model's training data.

Regarding adding the number of potential RBPs identified, we have added a sentence including the number together with a following sentence explaining the rationale:

L371-374: We used a conservative posterior probability threshold of 0.75 which retained 100 RBPs. This threshold was chosen to minimize false positives while accepting that some genuine RBPs with weaker sequence signatures might be missed.

• Line 363-368: clarity of this paragraph needs to be improved. The message is not clear. E.g., what does "genes involved in ... were mediated by RBPs ..." mean?

Response: We thank the reviewer for pointing out the need for more clarity here. We have thus modified the paragraph as follows:

L384-390: Interestingly, 00585 is among the top 10 most abundant proteins in *C. autoethanogenum*[13]. Most RBPs recognized cis-regulatory elements in both 5' and 3'UTRs of target genes while individual genes were rarely bound by RBPs in both of their UTRs. Within the latter, a potentially relevant example for autotrophy is the alcohol dehydrogenase 02655. While genes involved in coenzyme synthesis for redox reactions, quorum sensing, and base excision repair were regulated by RBPs exclusively via 3'UTRs, translation-related genes (e.g. amino acid metabolism, tRNA processing) and transporters showed binding events in both UTRs.

• Line 370: the number of 69 target genes showed be clearly named in the paragraph before to make clear from where this number comes from.

Response: We thank the reviewer for highlighting this shortcoming. We have thus modified a sentence in the preceding paragraph as follows:

L378-380: Overall, RBP binding sites were predicted for 69 differentially regulated genes: in 5'UTRs of ~11% of DTRs (20 genes) and ~15% of DTLs (27 genes), and in 3'UTRs of 12% of DTRs (21) and ~5% of DTLs (11) (Fig. 7; Supplementary Table 14).

• Line 387: change "by-products" to "products"

Response: We have made the change.

Discussion

• Should be streamlined in my opinion. It is now packed with information and discussion points. Reducing the information density together with increasing clarity in the language and sentence construction, would in my opinion be beneficial to convey the central messages of this study

Response: We thank the reviewer for pointing out this shortcoming. A similar comment on the need to improve readability was made by Reviewer #3. We have thus modified the Discussion and other parts to address both comments and to achieve better uniformity, decrease information density, improve wording clarity, and sentence construction. As these modifications are scattered around the whole manuscript, please see file 'Manuscript revised_with track changes' for changes.

• Line 410-411: this sentence suggests the possibility of widespread translational regulation, which the following sentences are arguing against. Suggest to rephrase this. The arguments presented from line 412-433 are in my opinion crucial for describing the regulatory network of acetogens (as far as it is known now) and should be expanded (see my comment above)

Response: We thank the reviewer for this comment.

Regarding the sentence in lines 410-411, we acknowledge that our wording was not clear. Our intention was not to state anything about our results but refer to the possibility in general that if an organism would display widespread translational regulation, this mechanism could play an important role in regulating

protein levels towards realizing the organism's phenotype. We have thus modified the sentence to clarify this point as follows:

L430-432: Widespread translational regulation in general could imply its dominant role in regulating protein levels towards realizing a cell phenotype.

Regarding the expansion of arguments presented in lines 412-433, we agree that it would be useful to expand the discussion. Considering that we added a new paragraph to Discussion as a response to a comment from Reviewer #3 (see comment '3. Regulatory network predictions…') and that addressing the previous comment from the reviewer made the Discussion longer, we judged that a limited expansion would be optimal here. This included adding a reference to a recent review that covers various aspects of the different regulatory layers of metabolism in acetogens, and should thus support the reader in achieving understanding of 'the big picture'. We thus modified the respective section as follows:

L432-456: We determined a low number of DTLs (186) that is consistent with the good correlation (R ~ 0.69 for 1,797 genes) between transcript levels quantified in syngas chemostats here and protein levels in LAbrini syngas chemostats in previous works[35,82] (Supplementary Fig. 11). The low DTL count is also in line with the low number of proteins differentially expressed between CO, CO+H_2, and CO+CO_2+H_2 grown *C. autoethanogenum* chemostats[108]. Indeed, metabolic flux adjustments between the latter cultures are dominantly accompanied by changing enzyme catalytic rates rather than concentrations[13]. Such post-translational regulation of fluxes is also seen in self-oscillating *C. autoethanogenum* autotrophic cultures[12,14] and this is presumably the most efficient regulatory mode of fluxes for energy-limited metabolism since close to half of ATP for biomass proliferation is used for protein synthesis[109,110]. Furthermore, keeping a higher baseline expression of key proteins would allow cells to use post-translational regulation for a more rapid reaction to gaseous substrates when these become available in natural environments. At the same time, nearly half of transcripts showed statistically different expression (q-value < 0.05) here (Fig. 2a; Supplementary Table 3), consistent with data from *C. autoethanogenum* syngas and fructose batch cultures[50]. Hence, transcriptional regulation seems to be required to ensure the baseline proteome expression for both autotrophy and heterotrophy. Our data thus suggest that *C. autoethanogenum* does not rely on strong or on/off regulation of genes key for facilitating stable growth on gas and sugar. This is in line with conclusions drawn in a recent review about different layers of regulation of metabolism in acetogens[3]. Still, transcriptional control of Rnf complex components and alcohol and aldehyde dehydrogenases detected in our work (Fig. 6) suggests attractive strain engineering targets in key metabolic activities of *C. autoethanogenum*.

Overall, ~19% of differentially regulated genes showed interactions with RBPs in our dataset (Fig. 7). The fact that we did not identify any RBPs binding only to genes with key activities for autotrophy (e.g. WLP, Rnf, Nfn, hydrogenases) implies that acetogens, at least *C. autoethanogenum*, do not seem to use an RBP-level mechanism for specifically regulating expression of genes exclusively needed for autotrophy. Thus, further studies are needed to understand the mechanisms behind post-transcriptional regulation of gene expression in acetogens[12-14].

• Line 467: Change "the most" to "the highest number of"

Response: We have made the change.

• Line 501-502: would tune that statement down since no interaction was determined which is directly linked with autotrophy. For this reviewer, this is the significant achievement of this study, elucidating that this type of regulation does not appear to play a major role in regulating acetogen metabolism.

Response: We agree with the reviewer and have modified the sentence as follows:

L538-539: Secondly, we report predicted genome-wide protein-RNA interactions for differentially regulated genes in a model-acetogen.

Methods

• Cultivation conditions: report gassing rates + agitation for syngas and heterotrophic conditions together in one sentence, and remove second reference to fructose concentration in the paragraph

Response: We thank the reviewer for this suggestion and have thus modified the sentence as follows:

L553-555: We used a syngas flow rate of 50 mL/min and agitation of 675 RPM for autotrophic, and a nitrogen flow rate of 50 mL/min and agitation of 200 RPM for heterotrophic cultures.

• Line 519-520: write "g/L biomass or cell dry weight" instead of "gram of dry cell weight per litre"

Response: We see that using 'g/L biomass' would be incorrect here as the reported values are for dry cell weight (not wet). We also believe that using just 'cell dry weight' would be incorrect as the reported values are concentrations, not absolute masses (which 'cell dry weight' would imply). We thus replaced 'dry cell weight' with 'cell dry weight' as seen below:

L555-558: With this, we achieved steady-state biomass concentrations of 1.37 ± 0.003 and 1.03 ± 0.008 gram of cell dry weight per liter for syngas and fructose cultures, respectively, for chemostats at dilution rates (D) 1.03 ± 0.05 and 0.997 ± 0.003 day^{-1} for syngas and fructose cultures, respectively.

Figures

Figure 2:

• describe cut-off for DTR in figure legend

Response: We thank the reviewer for highlighting this and have thus added the following sentence to the legend:

L155-157: Volcano plot showing genes determined as differentially transcribed (DTR; #176) as a subset of 1,784 genes with statistically different expression (q-value < 0.05) between syngas and fructose chemostats (DTRs are top 5% ranked according to q-value).

• the inset of figure 2b lacks a y-axis scale. Please insert.

Response: We thank the reviewer for pointing out this mistake. We have added the scale for the inset figure as seen below:

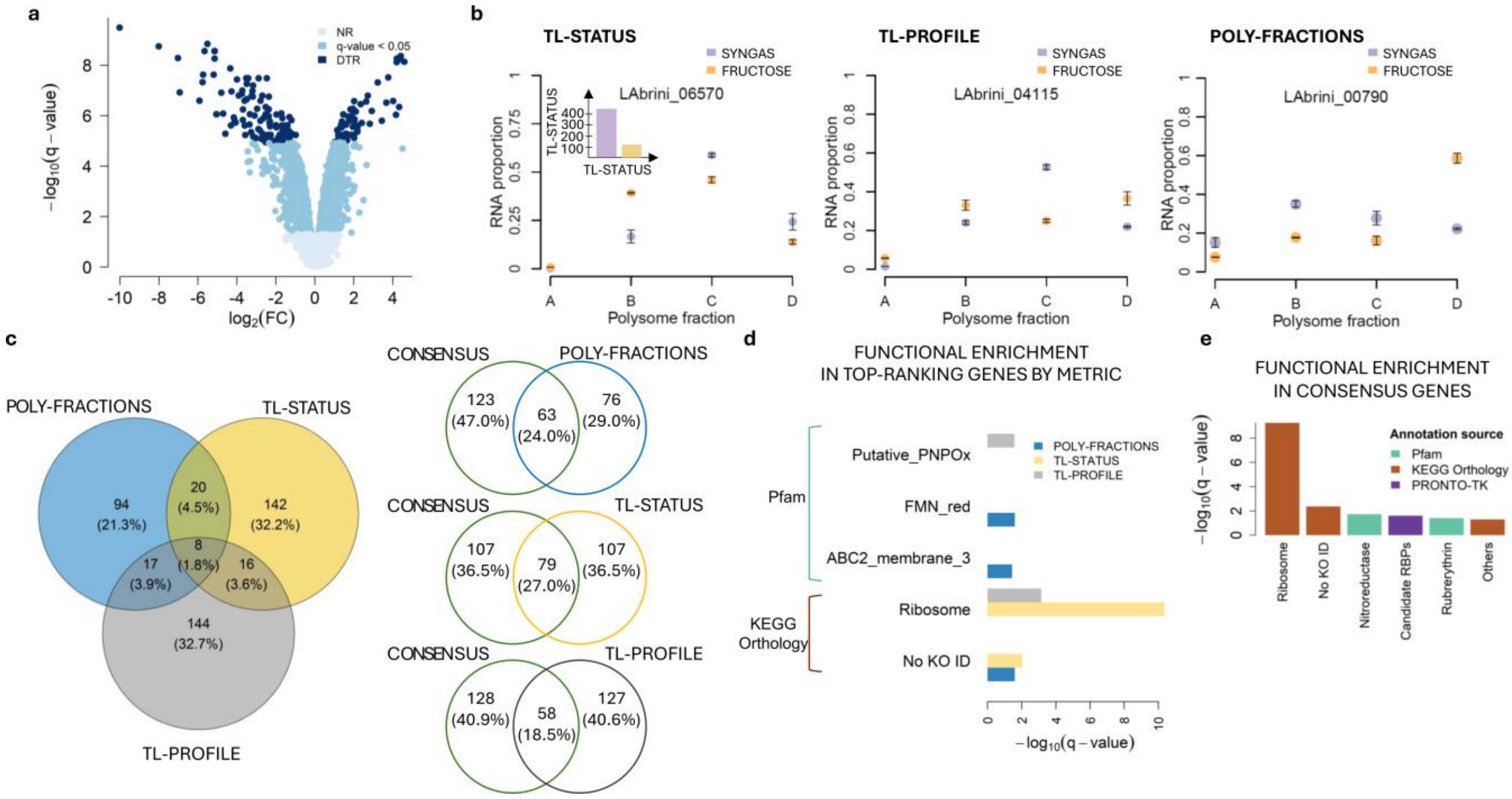

Figure 3:

• Color of PRONTO-TK in annotation source does not match color of “candidate RBPs” in Figure 3b

Response: We thank the reviewer for highlighting this mistake. We have changed the ‘Candidate RBPs’ color to orange to match the legend as seen below:

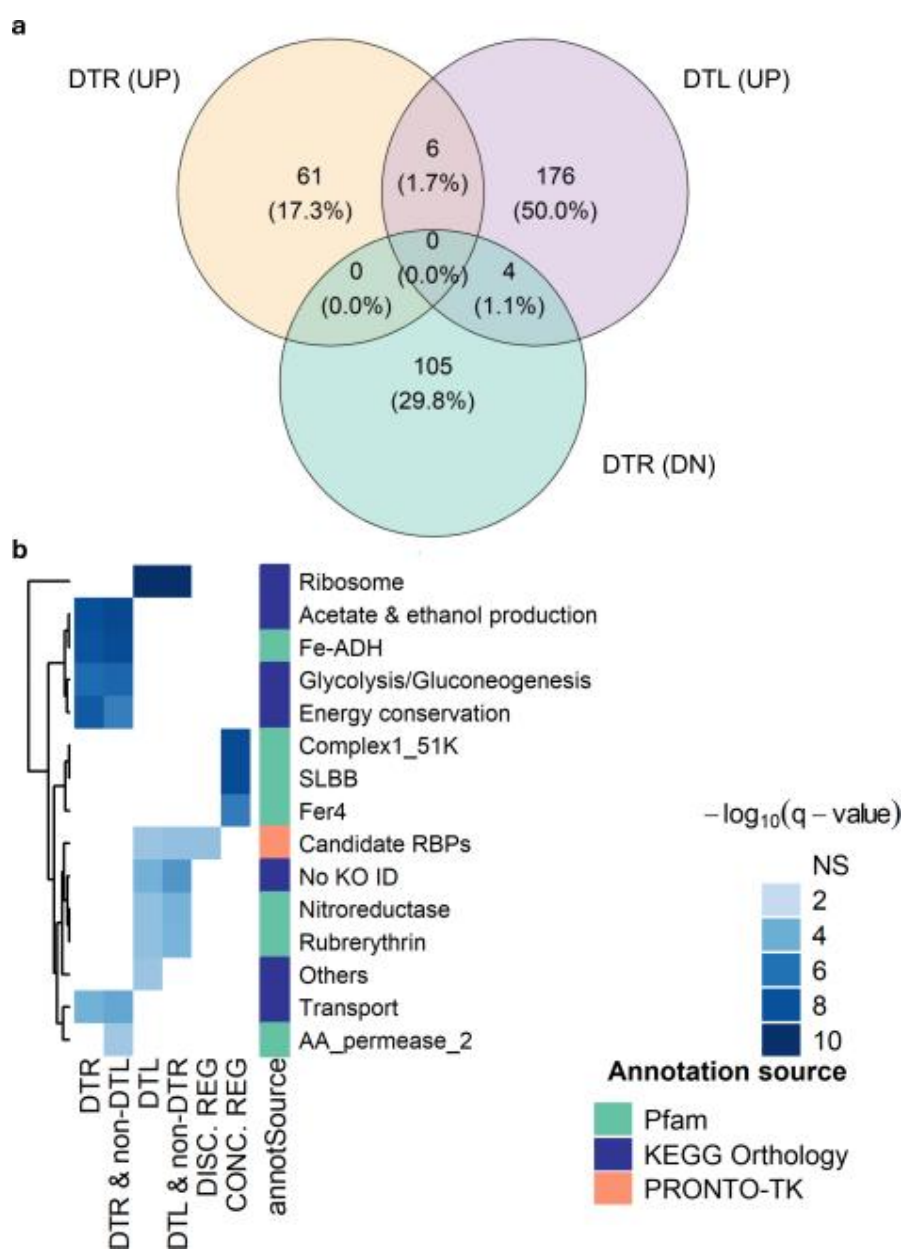

Figure 6:

• What is the central circle in the figure representing?

Response: We thank the reviewer for this comment. Our intent was just to illustrate the connectivity of pathways with the circle in the center of the figure but we agree that this could be potentially misleading. We have thus added an illustration of a bacterial cell within the circle to clarify this point as seen below:

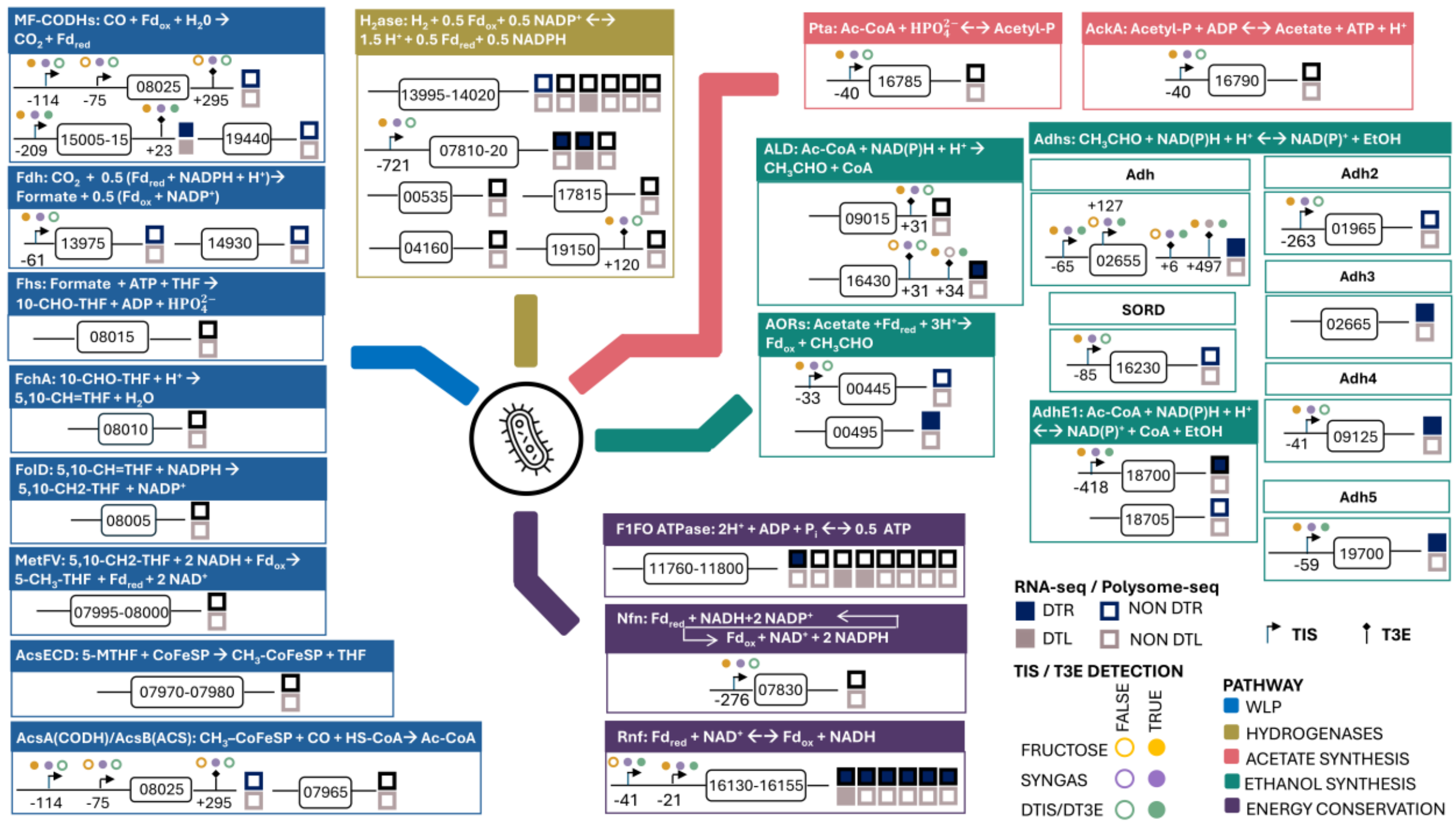

• The text legend should explain in more detail the symbol legend shown in the figure

Response: We thank the reviewer for pointing out the need for a more detailed legend. We have thus extended the description of symbols and abbreviations in the legend as follows:

Fig. 6. Transcriptional architecture and transcriptional/translational regulation in key metabolic pathways for autotrophy of *C. autoethanogenum*. Boxed numbers are LAbrini gene IDs. Transcription initiation (TIS) or transcription 3' end (T3E) site distance shown from start codon of downstream coding DNA sequence (CDS) or stop codon of upstream CDS, respectively. Color-coding of circles: open yellow or purple, TIS/T3E not detected for fructose or syngas; filled yellow or purple, TIS/T3E detected for fructose or syngas; open green, not differentially expressed TIS or T3E; filled green, differentially expressed TIS (DTIS) or T3E (DT3E). Color-coding of squares: filled blue, differentially transcribed gene (DTR); open blue, non-differentially transcribed gene (NON DTR); filled grey, differentially translated gene (DTL); open grey, non-differentially translated gene (NON DTL). MF-CODH, monofunctional carbon monoxide dehydrogenase; Fd, ferredoxin; THF, tetrahydrofolate; 10-CHO-THF, 10-formyl-THF; 5,10-CH=THF, 10-methenyl-THF; 5,10-CH2-THF, 10-methylene-THF; 5-CH3-TFH, 5-methyl-THF; CoFeSP, corrinoid iron sulfur protein; Ac-CoA, acetyl-CoA; EtOH, ethanol; SORD, zinc-dependent dehydrogenase. See Supplementary Table 1 for all gene descriptions. This figure has been designed using resources from Flaticon.com.

• SORD should be explained in the figure legend

Response: We thank the reviewer for highlighting this. We have added the gene description for SORD, please see our previous response.

• CoFeSP and THF should be explained in the legend

Response: We thank the reviewer for pointing this out. We have added definitions to these abbreviations, please see our response above.

Reviewer #2

The manuscript by Re et al presents a multi-omics study that combines chemostat cultivation, RNA-seq, and computational tools to investigate gene controls in the model acetogen Clostridium autoethanogenum. In addition to regular transcriptomic RNA-seq, the authors have conducted multiple variants of RNA-seq, including ribo-seq (polysome-seq) to analyze translating mRNAs, Cappble RNA-seq to identify transcription start sites, and Term-seq to identify transcription termination sites. This analysis generated global RNA maps of transcription start and termination sites, which could be a fine resource for RNA research in C. autoethanogenum. However, comparing the differential gene expression profile at two different growth conditions (syngas vs. fructose), the authors identified little changes that are of significance in the acetogen biology. The authors further attempted to predict potential RNA-binding proteins (RBP) in C. autoethanogenum, but failed to confirm any of the predicted RBP-RNA interactions and to demonstrate their contribution to autotrophy.

Response: We thank the reviewer for acknowledging the potential of our results being a fine resource for RNA research in *C. autoethanogenum*. We agree that our analysis did not identify many large changes between the two growth conditions. We feel that such a result itself is also relevant for a better understanding of acetogen metabolism, as pointed out by Reviewer #1 as being a significant and impactful outcome. Regarding the relevance of the predicted RNA-protein interactions for autotrophy, we have rephrased wording in all sentences which could have implied such links (please see our response to the comment of Reviewer #1 'Remove statement about involvement of RBP-level regulation of autotrophy'). We note that it could be very challenging to experimentally and unequivocally verify the relevance of our predicted RBP-RNA interactions due to the large number of candidate RBPs, their binding to genes with potentially essential functions but not involved in autotrophy directly, and the difficulties with creating such a number of *C. autoethanogenum* gene deletion mutants. This would be outside the scope of a single study using acetogens.

Specific comments are below:

- Line 50. No RBPs have been described in acetogens so far, but several known RNA-binding proteins have been characterized in its close-related relatives Clostridium diffcile, including various ribonucleases and Hfq (Fuchs et al, PNAS 2021, PMID: 34131082).

Response: We thank the reviewer for pointing out these references. We have thus modified the sentence as follows:

L48-50: While some RBPs have been characterized in Clostridia[32], no RBPs have been described in acetogens so far, and thus we also lack knowledge about RBP-RNA interactions.

We note that our study also predicted within RBPs Hfq and eight ribonucleases, five of which were found to regulate DTRs or DTLs (Supplementary Table 13), see also updated Fig. 7 below.

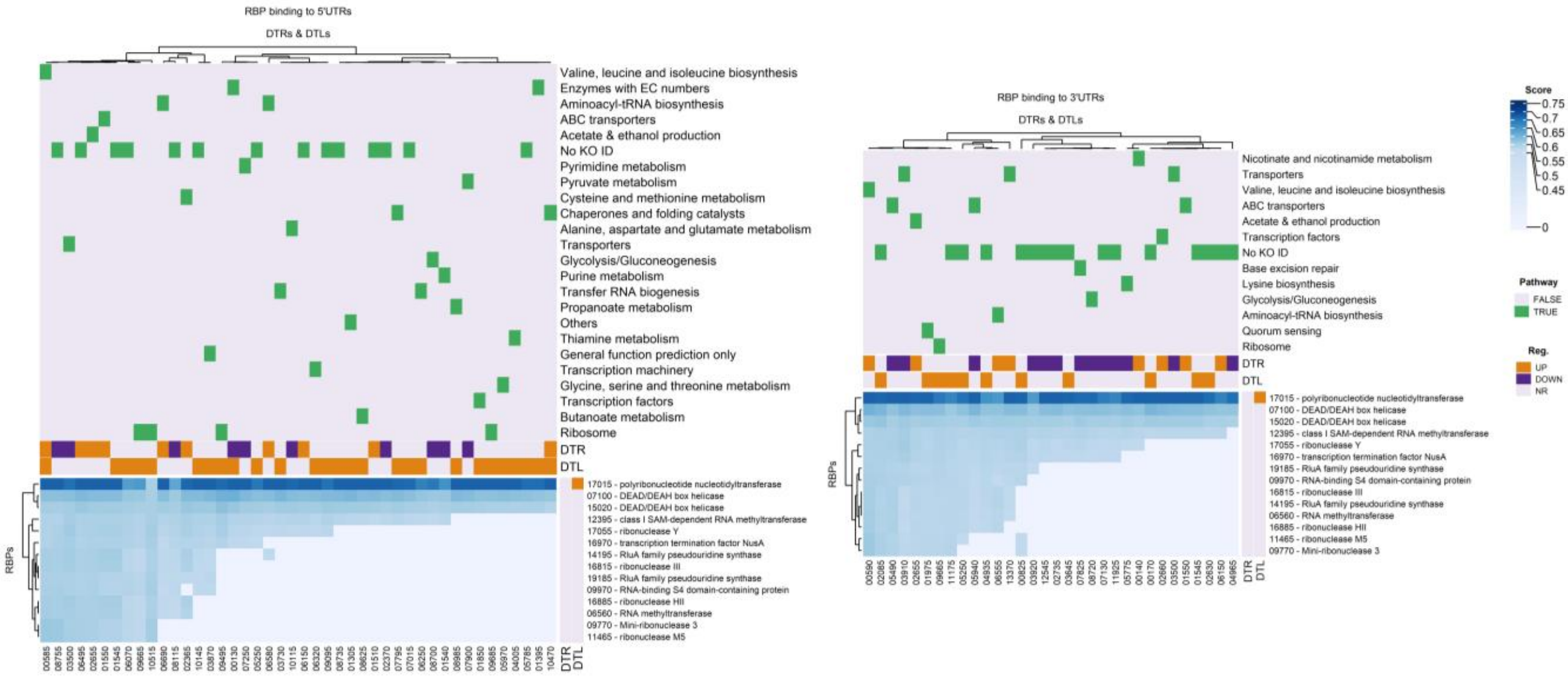

- Line 53, the aim of the work is to identify for the first time in acetogens RBP-RNA interactions. This is unfortunately not achievable by the experimental design, and not achieved by the data collected so far.

Response: In our opinion, results of a study do not invalidate the aim(s) of the study. In our case, we first aimed to experimentally determine RBPs contributing to autotrophy but without success. We then took another approach to predict RBPs relevant for autotrophy. Considering the comment from Reviewer #1 as well on this sentence, we have modified it as follows:

L53-54: The aim of our work was to identify genome-wide RBP-RNA interactions that contribute to autotrophy in acetogens.

- Line 129-143, The quality of the polysome-seq is unclear. Reads coverage are not shown for mRNAs to confirm CDS-only coverage by ribosomes, and for noncoding RNAs that are expected to be little bound to polysomes. It is also unclear whether these polysome-seq data are sufficiently quantitative for protein expression analysis.

Response: We thank the reviewer for this comment.

Regarding read coverage data. In a polysome-seq experiment, mRNA-ribosome complexes are fractionated according to the number of ribosomes bound to each mRNA. Several fractions are generated ranging from 0 ribosome (ribosome-free mRNAs) to ~8 ribosomes (polysomes). Within each fraction, ribosomes are subsequently removed from the mRNAs by protein precipitation, after which total RNA is extracted, and the cDNAs from corresponding mRNAs sequenced after library preparation. This approach provides information about the number of ribosomes loaded on each mRNA molecule (*i.e.* translational status), inferred from the fraction in which it is found. However, it does not provide information about the positions of ribosomes on the mRNA. Ribosome position can be determined by Ribo-seq from read coverage data but not by polysome-seq analysis. Therefore, we cannot compare read coverage between translated mRNAs and non-translated noncoding RNAs.

To validate our polysome-seq approach, we can first look at the raw read counts of non-translated RNAs in our polysome-seq data. For this, we analyzed three annotated ncRNAs (Labrini_06170, 06105, 13970), which are annotated by the NCBI pipeline without experimental evidence, one tmRNA (11030), and the SRP RNA (08445) of *C. autoethanogenum*. As shown in the figure below, four out of five were, as expected, more abundant in fraction A (non-translated RNAs) than in fractions B, C, or D (actively

translated RNAs) under both growth conditions. The higher abundance of ncRNA Labrini_06170 in fractions B, C, or D is likely due to hybridization with mRNAs undergoing translation.

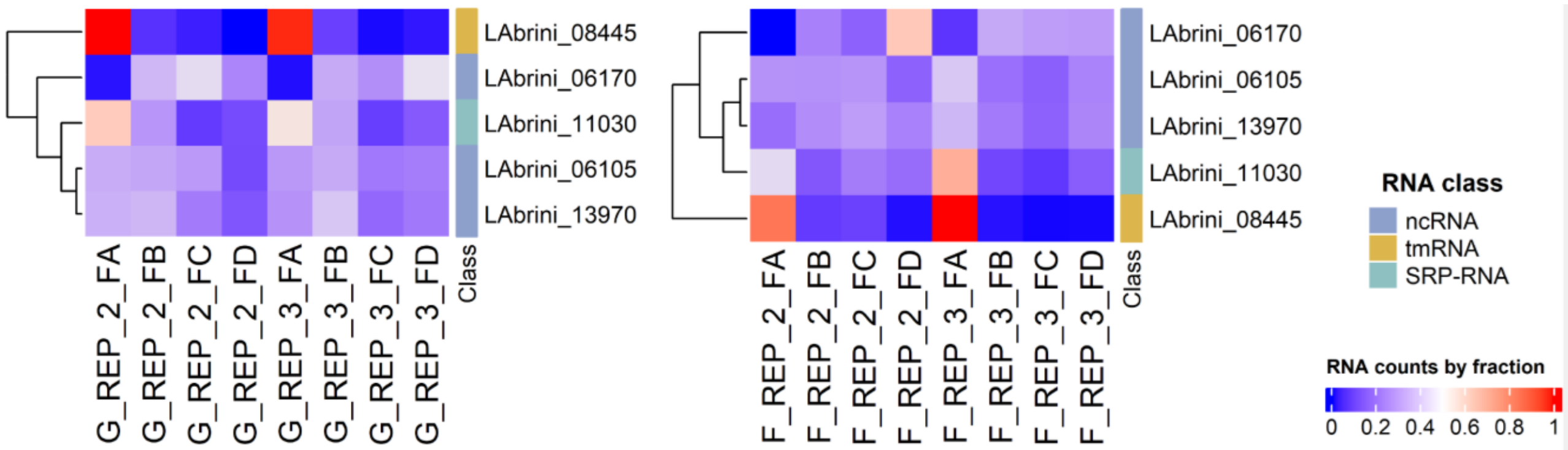

G, syngas; F, fructose; REP, bio-replicate.

Second, we have previously validated, across several bacteria, that polysome-seq correlates with translation efficiency in *Escherichia coli* (DOI: 10.1128/spectrum.02041-21) and with protein abundance in *Lactococcus lactis* (DOI: 10.1186/1471-2164-13-528). Indeed, we can observe a positive correlation between raw poly-seq data from fractions B+C+D (representing all translated RNAs) from this work and protein abundance in *C. autoethanogenum* during syngas growth in previous works (top row in figure below). This correlation supports the conclusion that polysome-seq experiments reliably reflect translational states, thereby confirming their relevance. Additionally, the strong correlation between raw poly-seq data from fractions B+C+D and transcript abundance from this work (bottom row in figure below) is consistent with the current understanding in the literature that translational regulation has a limited role in acetogens. This further supports the reliability of our polysome-seq data.

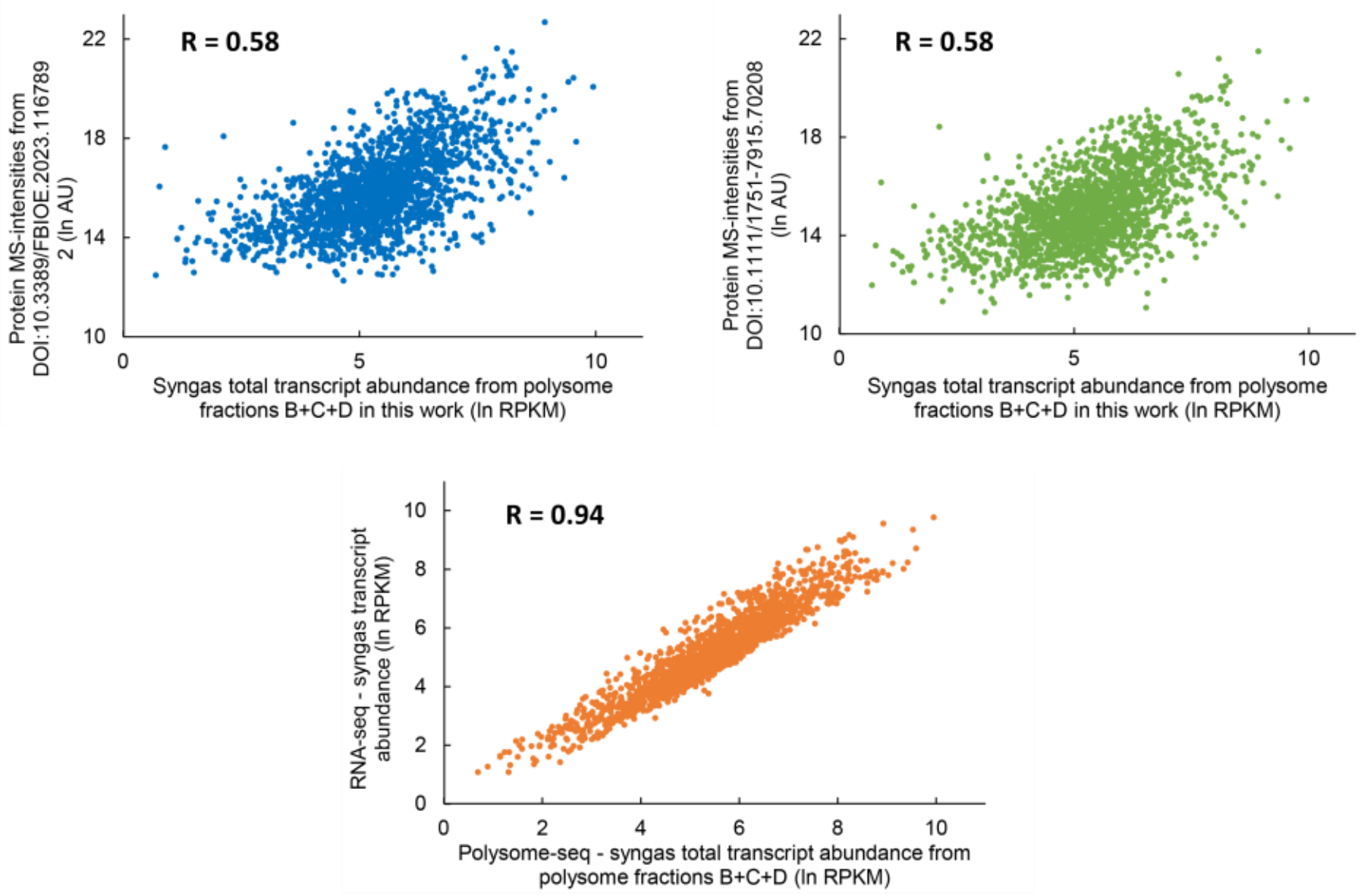

R, Pearson correlation coefficient

- Line 124-128, only the top 5% genes with the lowest q-value was considered as differential expression. Was fold-change even considered? Such strong suppression is unusual and introduces unwarranted bias and conclusions in data analysis, such as the coupling of transcriptional and translation control. The authors' view may be largely distorted from real world gene expression.

Response: We thank the reviewer for this comment.

The reviewer is correct in that the top 5% ranking genes from differential transcription analysis were determined only based on q-values, *i.e.* fold-change was not considered. We note that differential expression analysis datasets in general show a clear positive correlation between fold-changes and q-values if the experiments and –omics analysis are carried out appropriately. This is most easily seen from Volcano plots, in our case Fig. 2a. Thus, our top 5% cut based on q-values indirectly also considers fold-changes. Our top 5% cut is actually not 'stronger suppression than usual' as the lowest ranked gene within the top 5% set has a fold-change of ~0.5 (*i.e.* 2-fold repression on syngas vs fructose). Therefore, our top 5% cut based on q-values to determine DTRs selects genes similar to the often-used arbitrary fold-change threshold of >2 in differential expression analysis. Hence, in our opinion, our analysis does not introduce 'unwarranted bias and conclusions in data analysis'.

Regarding the effect of our criteria for determining DTRs on the conclusions about coupling of transcriptional and translational control, we also see that our approach does not distort our conclusions. This is supported by the fact that if we vary the top-ranking gene %-threshold between 1 and 10% for both DTRs and DTLs, the proportion between coupled/uncoupled genes changes minimally (left plot on figure below; coupled changes are in yellow). Thus, our conclusion that the large majority of expression changes are transcriptionally and translationally uncoupled holds within a 10-fold %-threshold range. The density plot in the center of the figure below also illustrates indirectly the positive correlation between q-values and fold-changes in transcript expression data. The density plot on the right shows the correlation between the %-threshold cut for DTLs and distribution of values for one of the three metrics used (TL-STATUS) to evaluate translational regulation. We note that we specified the definition of coupling in the revised manuscript as requested by the reviewer in a comment below to be '*If we define transcriptional and translational regulation being coupled when a gene shows homodirectional expression changes as a DTR and DTL,…*'.

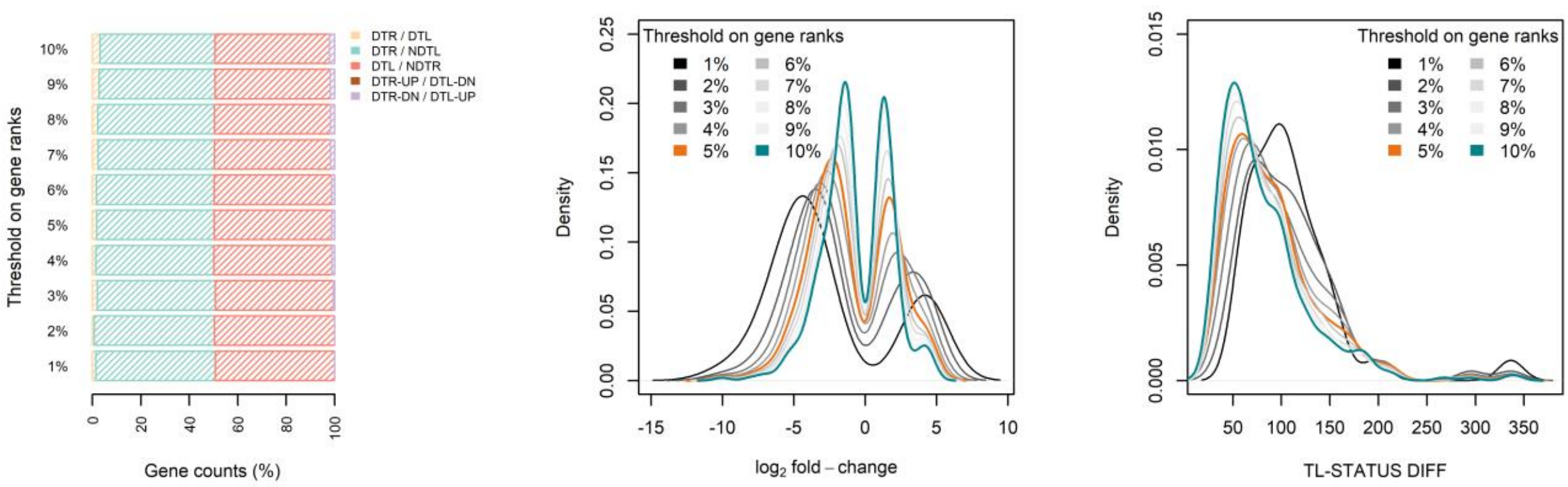

Left, barplot showing the relative frequency of coupled (DTR/DTL; yellow) and uncoupled genes (all others) at multiple %-thresholds used to select genes. N, non; DN, down. **Centre**, density plot showing the distribution of log_2 fold-changes in transcript levels of the genes retained at each %-threshold. **Right**, density plots showing the distribution of TL-STATUS metric values of the genes retained at each %-threshold.

Additionally, we assessed whether the functional characterization of transcriptional/translational changes between syngas and fructose could be affected by a sharp threshold to define DTRs and DTLs. In the manuscript, enrichment analysis of functional classes was performed using overrepresentation analysis (*i.e.* one-sided hypergeometric test at FDR = 5%; see Fig. 3b), which inherently relies on a sharp threshold to define DTRs and DTLs. As a response to this comment, we used also the gene set enrichment analysis (GSEA) scoring method for functional classes (DOI: 10.1038/ng1180) as implemented in the package fgsea in the software R. We used GSEA since it does not require a user-set threshold on genes, but it rather takes as input a gene list sorted by a metric of differential expression. Here, we applied fgsea to genes ordered by the product between $\log_2$ fold-change (LFC) and $-\log_{10}$ q-value for differential transcriptional analysis and to genes ordered by the rank in CONSENSUS approach for differential translational analysis. Comparison of enrichment analysis results from the overrepresentation analysis reported in the manuscript (see Fig. 3b) and GSEA here (figure below) shows that both methods provide consistent outcomes. Thus, in our view, our analysis does not introduce bias towards the conclusions about enriched functional classes in DTR and DTL datasets.

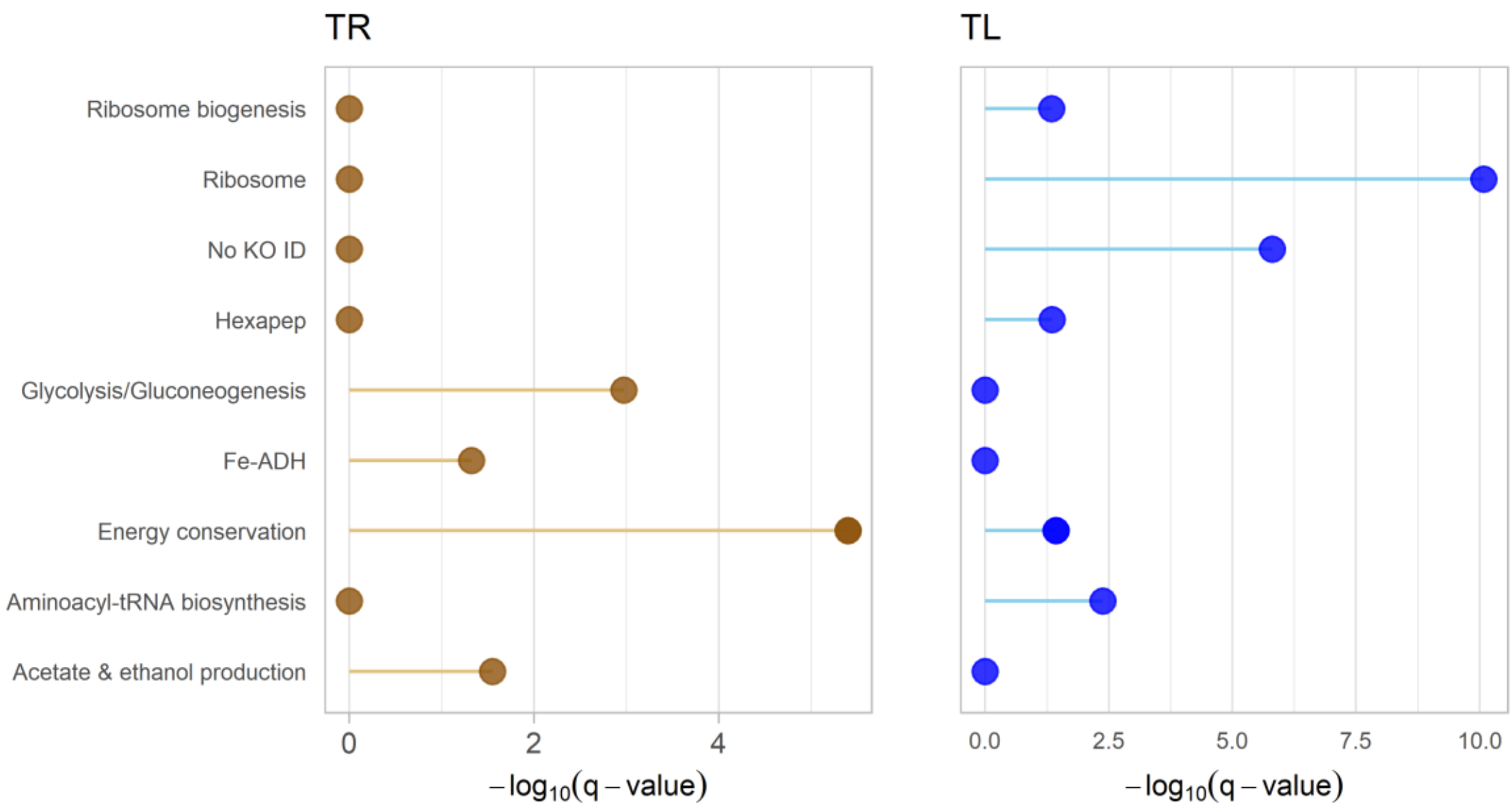

TR, transcription; TL, translation

- Figure 2, Limited transcriptional and translation control. Is the control really limited or suppressed in data analysis?

Response: We believe the use of 'limited control' in the figure title is reasonable. To the best of our knowledge, good practice for analysis of differential expression data involves setting thresholds. We decided to use the same threshold (i.e. top 5% ranking genes) for determining DTRs and DTLs to ensure consistency for data integration and combined analysis.

For RNA-seq data, we detected 1,784 genes with statistically differential expression (q-value < 0.05) between syngas and fructose growth (see Fig. 2a; Supplementary Table 3). If we were to set the often-used arbitrary fold-change threshold of >2 for these genes, 528 genes would be considered differentially transcribed. This number is larger than our 176 for DTRs, but both, in our opinion, are a small fraction from the 4,043 annotated genes in LAbrini. Thus, transcriptional control seems to be limited. For polysome-seq data, we intended to prioritize detection of stronger and statistically more significant translational differences across the three distinct metrics to potentially capture different regulatory layers of translational (TL-STATUS, TL-PROFILE, and POLY-FRACTIONS). The use of a single threshold to

determine DTLs other than a ranking-based one (*e.g.* fold-change) would not be possible in our case as the calculated metrics yield variable quantifiers: absolute difference in translation status for TL-STATUS; a relative difference between the polysome profiles for TL-PROFILE; and absolute number of fractions for POLY-FRACTIONS. Based on this, we considered the fraction of 186 genes determined as DTLs from the 3,897 annotated CDSs in LAbrini as minor, and thus translational control limited.

We see that our conclusion is consistent with the literature as described in Discussion:

L432-440: We determined a low number of DTLs (186) that is consistent with the good correlation (R ~ 0.69 for 1,797 genes) between transcript levels quantified in syngas chemostats here and protein levels in LAbrini syngas chemostats in previous works[35,82] (Supplementary Fig. 11). The low DTL count is also in line with the low number of proteins differentially expressed between CO, CO+H_2, and CO+CO_2+H_2 grown *C. autoethanogenum* chemostats[108]. Indeed, metabolic flux adjustments between the latter cultures are dominantly accompanied by changing enzyme catalytic rates rather than concentrations[13]. Such post-translational regulation of fluxes is also seen in self-oscillating *C. autoethanogenum* autotrophic cultures[12,14] and this is presumably the most efficient regulatory mode of fluxes for energy-limited metabolism since close to half of ATP for biomass proliferation is used for protein synthesis[109,110].

- Line 173 and Figure 3, Uncoupled transcriptional and translational control. This is overstatement and inaccurate. The exact concept of coupling is not defined in the manuscript, and lacks well-defined controls.

Response: We thank the reviewer for pointing out the shortcoming of not defining what we mean by coupling between transcription and translation. We have now added the definition and also new results as a response to a comment from Reviewer #3 (see comment '2. A similar analysis as…') that support the conclusion that our data show that transcriptional and translational control seem uncoupled under these conditions. Please also refer to our previous two responses on this matter.

L176-182: This very low ~2% overlap (6 genes from 352) at gene-level is consistent with a weak agreement between highly ranking gene sets grouped based on q-values or CONSENSUS ranks from differential transcriptional and translational analysis, respectively (Supplementary Fig. 6). If we define transcriptional and translational regulation being coupled when a gene shows homodirectional expression changes as a DTR and DTL, our data suggest that transcriptional and translational regulation involved in realizing autotrophy in *C. autoethanogenum* are uncoupled.

- Line 155-156, translation genes are strongly enriched in DTLs. Why is this reasonable and expected?

Response: We thank the reviewer for this comment. We found that the reference was incorrect and the sentence was inconsistent. Regarding DTLs being enriched in translation-related genes, the expectation arises from the fact that ribosomal proteins are known to exert self-regulation by autogenous repression. We have now added the correct reference and modified the sentence as follows:

L151-153: Strong enrichment of translation-related genes in DTLs (Fig. 2e) is consistent with ribosomal proteins exerting self-regulation by autogenous repression[57].

- Although the authors have mapped thousands of TIS and T3S, few are different between syngas and fructose conditions. Nothing new has been learned by doing such analysis.

Response: We believe that the result of detecting few genes that use different TISs and T3Es between heterotrophic and autotrophic growth is also relevant for a better understanding of acetogen

metabolism, as also pointed out by Reviewer #1 as being a significant and impactful outcome. We see our dataset also being a valuable resource for the community, as also pointed out by Reviewer #3. Experimental determination of TISs and T3Es provides actual transcriptional boundaries compared to genome annotations derived from bioinformatic analysis. This is relevant for further investigations of 5'UTR and 3'UTR regions, a better description of operon structures, genetic construct design for metabolic engineering etc. To facilitate convenient use of our transcriptional architecture dataset for anyone interested, we provide Supplementary Table 9 that contains coordinates and annotations of CDSs, TISs, and T3Es for easy visualization with any relevant tool, *e.g.* the open-source Integrative Genomics Viewer. We find this very useful for our research in general since at least recently NCBI did not support supplementing their genome annotation information with experimentally determined transcript boundaries. This likely discourages readers and other researchers interested in the data to use this information in their work.

- Line 346, this entire section of RBP analysis is highly speculative. The predicted candidate RBPs are mostly known enzymes in RNA metabolism. PRONTO-TK is a prediction software based on function annotations, which lacks the power to identify new RNA-binding proteins. None of the predicted RBP or RBP-RNA interactions has been experimentally verified, adding little to the relevant field.

Response: We understand that one could see our RBP predictions being highly speculative. In the revised manuscript, we have modified sentences throughout the text to make it clear that all our RBP and RBP-RNA interaction results are computational predictions, not experimental identifications (please see revised manuscript with track changes). Hence, we now feel that our results are presented in a fair way, allowing the reader to evaluate their nature adequately. As pointed out by the reviewer, our predicted RBPs include well-known enzymes in RNA metabolism, thus supporting the reliability and relevance of our predictions.

We believe there is value in applying PRONTO-TK for RBP prediction. This is because PRONTO-TK approach leverages cutting-edge, language model (LM)-based representations of biological sequences to generate predictions for protein function in any organism. Natural language models have recently shown their enormous potential in filling the sequence-annotation gap by generating descriptive representations, *i.e.* embeddings, for the steadily growing number of proteins with unknown function from just their sequences and overcoming the limitations of standard feature-based machine learning methods. LM representations can be used as input to subsequent supervised training, thereby transferring knowledge acquired during task-agnostic, self-supervised pre-training on massive amounts of proteins to the task of predicting specific aspects of protein functionality.

Regarding lack of RBP verification, we note that it could be very challenging to experimentally and unequivocally verify the relevance of our predicted RBP-RNA interactions due to the large number of candidate RBPs, their binding to genes with potentially essential functions but not involved in autotrophy directly, and the difficulties with creating such a number of *C. autoethanogenum* gene deletion mutants. This would be outside the scope of a single study using acetogens. We agree with Reviewer #1 and #3 that our work is relevant for several fields as it integrates RBP predictions with experimentally mapped transcriptional architecture to provide a systematic reconstruction of the potential post-transcriptional regulatory interactions in a model-acetogen and offers valuable targets for follow-up experimental studies. We also note that we supply all raw data with the manuscript, thus interested researchers can re-analyze the data, investigate all predicted RBPs and RBP-RNA interactions, design follow-up validation experiments etc.

- Most figures are too abstract, showing little results and real data.

Response: In our opinion, most figures show results and real data from our experimental or computational work.

From the seven main text figures, only two – Fig. 1 and Fig. 4 – do not present either data or results from our work. We believe that both figures support the text for an easier understanding of the research done in this work. We feel that Fig. 1 is useful for such a systems-level study as it visually summarizes all key experimental and computational approaches used in our work for guiding the reader through the manuscript. To the best of our knowledge, such 'study summary' figures are common in systems-level studies where multiple datasets are integrated and further processed. We see that Fig. 4 is valuable to support the understanding of all the steps we took for the identification of transcription initiation sites and transcription end sites. We feel that a clearer description of this heuristic procedure is important for the reader to assess the reliability of our transcriptional architecture data. The whole procedure could be potentially challenging to fully comprehend by reading just the Methods text.

From the 11 supplementary figures, only the new figure added for this revised manuscript (Supplementary Fig. 8) to address a comment from Reviewer #3 does not present data or results. We agree with Reviewer #3 that the new figure supports easier understanding of main text Fig. 5 panels e and f.

Reviewer #3 (Remarks to the Author):

Re et al. present a manuscript in which they collect multi-omics data on translation and RNA expression in the model acetogen Clostridium autoethanogenum, and then apply sophisticated computational analyses to identify the major drivers of gene-expression changes between autotrophic and heterotrophic growth conditions. They show that transcriptionally regulated genes and translationally regulated genes overlap only minimally, suggesting a decoupling of translational control from RNA-level control. In addition, they map 5' and 3' transcript ends and examine condition-dependent changes in end-site usage. Finally, they use one computational tool to predict RNA-binding proteins (RBPs) and a second tool to predict RBP binding to differentially regulated genes. Overall, I think this work will be a valuable resource for the bacterial community, especially researchers interested in acetogens. It should also be of interest to the broader gene-expression regulation community, as it provides a useful blueprint for integrating multiple gene-expression datasets and applying emerging tools to infer potential regulatory circuits. I consider the manuscript a strong candidate for publication, but I do think a few issues should be addressed prior to acceptance.

Response: We thank the reviewer for acknowledging the value of our work. We have addressed all the valuable comments raised by the reviewer, as detailed below, which has considerably improved the quality of the manuscript in our opinion.

Major points

1. Limited overlap between methods in the DTL analysis.

In the DTL analysis, the authors compare three approaches for identifying translationally regulated genes. They note that the overlap of regulated genes across methods is minimal and argue that this reflects important differences between the analyses. I would encourage a more cautious interpretation. Differential regulation depends on thresholds that are necessarily somewhat arbitrary, and many genes may fall just above or below a cutoff depending on the method. Rather than focusing only on the subset that passes a hard threshold, it would be informative to compare the distributions of scores/effect sizes across methods. For example, for genes called differentially regulated by TL-status, where do they fall in the percentile distribution when genes are ranked by TL-profile? Is there a significant shift relative to random expectation? If there is, then the methods may be capturing similar trends even if they disagree on which genes cross the cutoff.

Response: We thank the reviewer for pointing out the need for a more cautious interpretation of these observations. We agree that comparison of distributions across the three metrics is valuable and have thus performed such an analysis, as described below and included in Supplementary Methods in the revised manuscript. This indeed confirmed the reviewer's expectation: despite the limited overlap within the top 5% gene sets of the three metrics at gene (Fig. 2c) and functional class (Fig. 2d) levels, cross-comparison of these gene sets across the three metrics showed that all three capture similar trends in translational regulation. We thus modified our interpretation of the results from the three metrics in the text as follows and included a new Supplementary Figure visualizing the results from this analysis (Supplementary Fig. 5; see below):

L143-147: Despite these limited overlaps at gene and functional class levels, cross-comparison of the top 5% gene sets across the three metrics showed that all three capture similar trends in translational regulation (Supplementary Methods; Supplementary Fig. 5). Therefore, we decided to determine DTLs using a CONSENSUS approach: firstly, we calculated for each gene its average rank across the three metrics; and secondly, we ranked these average ranks and defined DTLs as the genes in the top 5%.

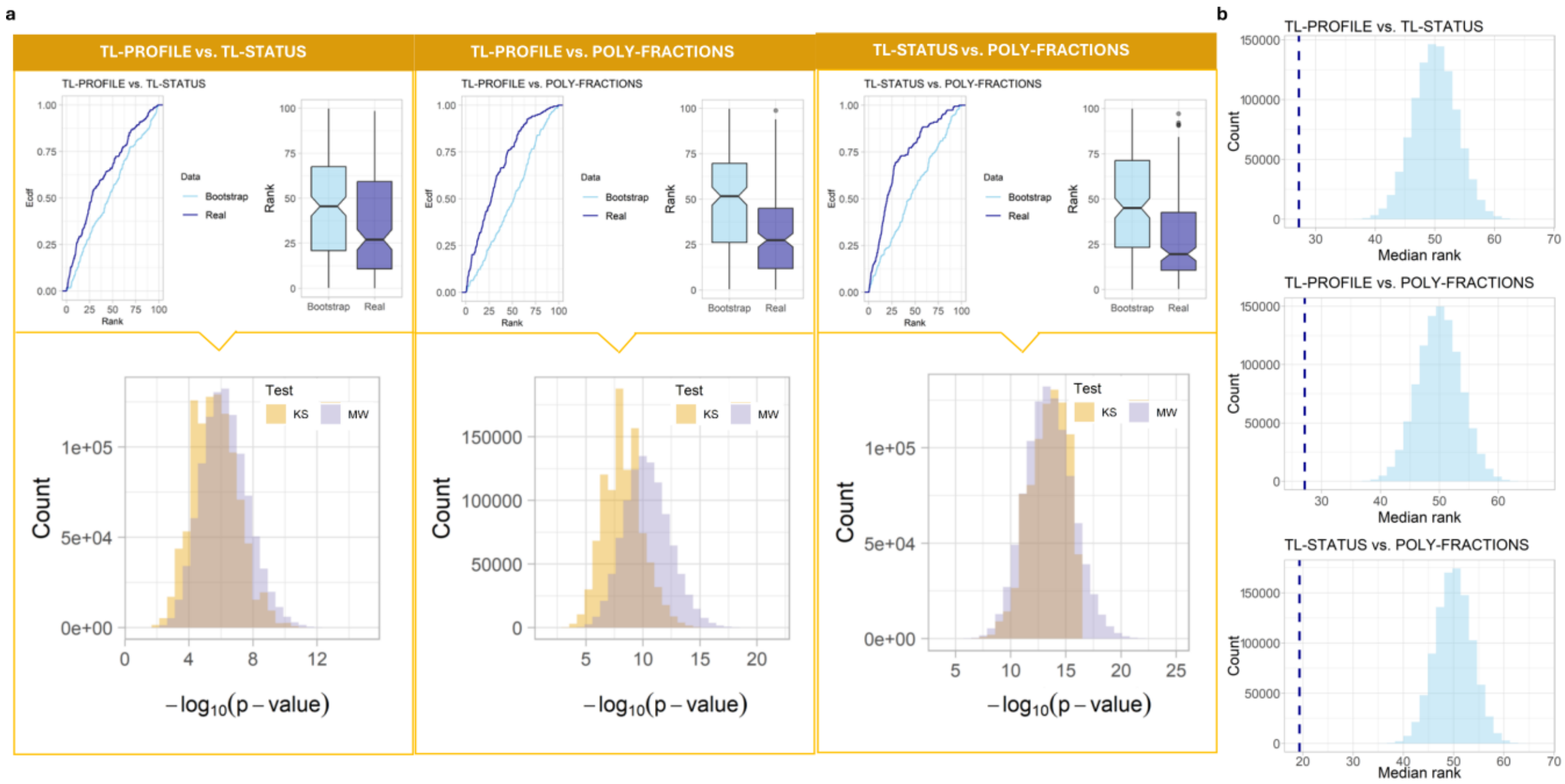

Supplementary Figure 5. Cross-comparison of top 5% gene sets across the three metrics used to evaluate translational regulation. a Analysis results from Approach A (see Supplementary Methods text for details). Top row shows differences in the empirical cumulative distribution function and central tendency between the ranks of the top 5% gene set ('Real') and randomly sampled ranks ('Bootstrap'). Bottom row shows $-\log_{10}$ p-value distributions. Ecdf, empirical cumulative distribution function; KS, Kolmogorov-Smirnov test; MW, Mann-Whitney test. **b** Analysis results from Approach B (see Supplementary Methods text for details). Median rank null distribution from random sampling is shown in light blue and median normalized rank from experimental data is shown as dashed dark blue line.

We also provide here the methodology we used for this analysis (same text as in Supplementary Methods):

We used two approaches to compare the distributions of gene ranks across the three metrics we used to evaluate translational regulation.

Approach A: Here, we normalized gene ranks to the range [0, 100] according to each of the metrics that we used to evaluate translational regulation (TL-STATUS, TL-PROFILE, and POLY-FRACTIONS). Firstly, we determined the normalized ranks that the top 5% ranking genes by a certain metric obtained by using another metric. Secondly, we evaluated if this rank distribution is significantly different from a rank distribution obtained by random sampling of gene ranks without replacement from a uniform distribution in the interval [0,100]. For this, we used both the Mann-Whitney (MW) test, which detects differences in central tendency, and the Kolmogorov-Smirnov (KS) test, which is sensitive to any difference in the distribution shapes. Since the statistical significance of the test result can vary according to the randomly sampled rank distribution, we repeated both tests 10^6 times to assess their range of variability. We carried out his procedure for each pair of the three metrics. Comparing the ranges of obtained p-values (see above for Supplementary Fig. 5a) showed that the top 5% gene sets from any of the three metrics are ranked statistically significantly higher than randomly sampled gene rankings when evaluating their rankings in the ranking obtained by another metric (significance threshold p-value 0.05; i.e. $-\log_{10}$(p-value) ~1.3).

Approach B: Identical to Approach A, we normalized gene ranks to the range [0, 100] according to each of the metrics that we used to evaluate translational regulation (TL-STATUS, TL-PROFILE, and POLY-FRACTIONS). Firstly, we determined the normalized ranks that the top 5% ranking genes by a certain metric obtain by using another metric. Secondly, we considered the median value of these ranks for

each metric as the test statistic and evaluated if it significantly differs from random expectations. For this, we constructed the null distribution of the test statistic by computing its value for each of the 10^6 random rank distributions that were obtained by random sampling a number of ranks equal to the number of top 5% ranking genes from a uniform distribution on the interval [0, 100]. We carried out his procedure for each pair of the three metrics. The empirical p-value was then obtained as the fraction of cases that produce a test statistic at least as low as that calculated for experimental data. This analysis showed that the median rank of top 5% ranking genes of any of the three metrics is statistically significantly lower than that of randomly sampled genes in the ranking obtained by another metric (see above for Supplementary Fig. 5b; p-value < 10^{-6}).

2. A similar analysis as just mentioned above would also help clarify the apparent overlap (or as pointed out by the authors the current lack thereof) between DTR and DTL gene sets.

Response: We thank the reviewer for this suggestion and agree that here as well a global comparison of DTR and DTL gene sets is valuable. We thus performed such analysis by ranking genes according to their q-values in differential transcription analysis and according to their ranks from the CONSENSUS approach in differential translation analysis. Analysis of gene counts across bins of ranks (Supplementary Fig. 6 below) showed weak agreement in highly ranking genes between the two analyses in line with the low gene-level overlap described in the original manuscript. We included this result in the revised manuscript as new text and as a new Supplementary Fig. 6:

L176-179: This very low ~2% overlap (6 genes from 352) at gene-level is consistent with a weak agreement between highly ranking gene sets grouped based on q-values or CONSENSUS ranks from differential transcriptional and translational analysis, respectively (Supplementary Fig. 6).

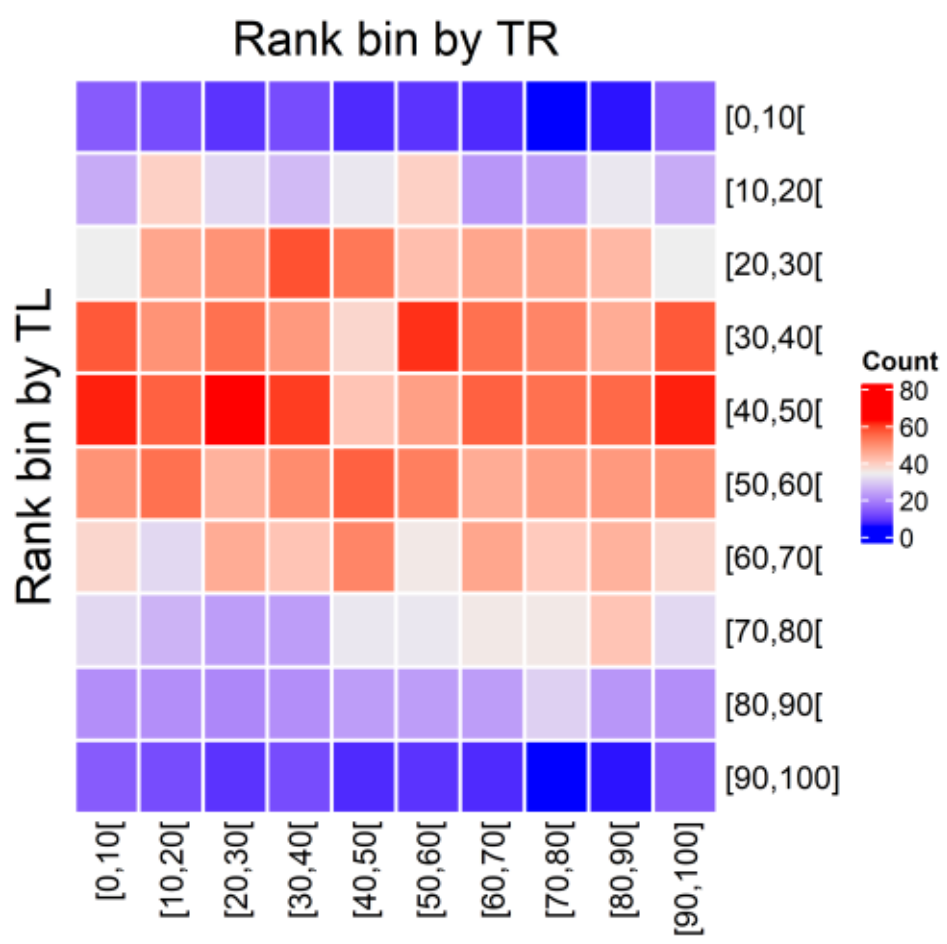

Supplementary Figure 6. Weak agreement in gene rankings by differential transcription (TR) and translation (TL) analyses. The heatmap displays gene counts by bins of ranks that genes obtained in analysis of differential TR (ranks based on q-values) and differential TL (ranks based on ranks from the CONSENSUS approach). Ranks are scaled to the interval [0, 100].

3. Regulatory network predictions are discussed too definitively.

A major concern with the Discussion is that the inferred regulatory networks are sometimes described in a way that reads as if the interactions are established. However, these networks are predictions produced by multiple computational steps (e.g., an algorithm to predict RBPs and another to predict binding sites/targets). The authors do not present experimental validation demonstrating that these interactions occur in vivo. The Discussion should more clearly reflect the predictive nature of these results and more explicitly discuss limitations, sources of uncertainty, and potential false

positives/negatives in the computational pipeline. I am not suggesting that experimental validation is required for publication, but a more balanced discussion - particularly one that more transparently frames these networks as hypotheses - would help readers interpret the predictions appropriately.

Response: We thank the reviewer for this valuable comment.

We agree that some of our wording could have been misleading in terms of the origin of the presented RBPs and RBP-RNA interactions. In the revised manuscript, we have modified sentences throughout the text (including Discussion) to make it clear that all our RBP and RBP-RNA interaction results are computational predictions, not experimental identifications (please see revised manuscript with track changes). Hence, we now feel that our results are presented in a fair way, allowing the reader to evaluate their nature adequately.

We acknowledge our shortcomings of not discussing limitations and sources of uncertainty of our computational pipeline and agree with the reviewer that this needs to be added. We thus added the following paragraph to Discussion with key points on these aspects (see below for a longer discussion). We also added two sentences to Results highlighting potential implications of the filtering criteria we used for both computational approaches (see below).

Paragraph added to Discussion:

L522-533: Our *in silico* reconstruction of RBP-mediated regulatory interactions has limitations as it relies on two computational tools that both involve predictive uncertainties. For instance, PRONTO-TK predictions could be limited by the underlying training data that are biased towards well-studied organisms and could potentially miss RBPs with non-canonical binding domains or, more generally, yet-to-be-elucidated regulatory mechanisms in non-model organisms. In addition, the catRAPID sequence-based algorithm cannot fully account for the dynamic cellular environment, including competitive protein binding, RNA conformational changes, or post-translational modifications that influence RBP activity *in vivo*. Nevertheless, we employed several design choices to minimize computational artifacts. Firstly, conservative filtering was adopted to minimize false positives in predicted RBPs while accepting that some genuine RBPs could be missed. Secondly, the requirement for convergent evidence from multiple scoring metrics reduced the likelihood of spurious predictions of RBP-RNA interactions. Overall, our computational predictions should be interpreted as well-founded hypotheses that require future experimental validation.

Sentences added to Results:

L371-374: We used a conservative posterior probability threshold of 0.75 which retained 100 RBPs. This threshold was chosen to minimize false positives while accepting that some genuine RBPs with weaker sequence signatures might be missed.

L376-378: We prioritized high-confidence predictions by retaining interactions ranking in the top 25% of catRAPID scores, which could potentially exclude weaker but functionally relevant interactions.

We provide here a longer discussion on the potential limitations and sources of uncertainty of our computational pipeline.

Our computational approach integrates established methodologies but necessarily involves predictive uncertainties that merit discussion. PRONTO-TK employs the ProtT5-XL protein language model, which

represents the current state-of-the-art in protein sequence analysis, capturing complex biochemical and structural relationships through transformer-based embeddings trained on millions of protein sequences. However, the underlying training depends on experimentally validated UniProt annotations, which may exhibit taxonomic bias toward well-studied organisms and could potentially miss RBPs with non-canonical binding domains or yet-to-be-elucidated regulatory mechanisms in non-model organisms. Our selection of a conservative posterior probability threshold of 0.75 was designed to minimize false positives while accepting that some genuine RBPs with weaker sequence signatures might be excluded from analysis.

The catRAPID predictions integrate multiple validated scoring criteria, including interaction propensity, RBP-specific binding potential, and recognition of established RNA-binding motifs. While this multi-parametric approach strengthens prediction reliability, sequence-based algorithms cannot fully account for the dynamic cellular environment, including competitive protein binding, RNA conformational changes, or post-translational modifications that influence RBP activity *in vivo*. Our decision to retain only interactions ranking in the top 25% of scores represents a stringent filtering approach that prioritizes high-confidence predictions while potentially excluding weaker but functionally relevant interactions.

The sequential nature of our computational pipeline, progressing from RBP identification through binding site prediction, compounds individual tool uncertainties in ways that are difficult to quantify precisely. Nevertheless, several design choices minimize computational artifacts: the use of experimentally determined UTR boundaries rather than predicted sequences provides accurate target regions for binding analysis; the integration of condition-specific transcriptional and translational data focuses predictions on biologically relevant targets; and the requirement for convergent evidence from multiple scoring metrics reduces the likelihood of spurious predictions. These computational predictions should therefore be interpreted as well-founded hypotheses that require experimental validation, with the systematic nature of our approach enabling rational prioritization of candidates for mechanistic follow-up studies.

4. Directionality of DTL changes (all upregulated in syngas).

For the DTL genes, why are all (or nearly all) upregulated in syngas relative to fructose? This pattern raises the possibility of a normalization or scaling issue, or alternatively a global shift in translation capacity/efficiency between conditions. The authors should address this explicitly and discuss potential technical and biological explanations.

Response: We thank the reviewer for the interesting question. In our opinion, the result of all DTLs being up-regulated on syngas is very unlikely caused by a technical/normalization/scaling issue. In our polysome-seq approach, mRNA-ribosome complexes are fractionated according to the number of ribosomes bound to each mRNA. In all the different fractions from both syngas and fructose, RNAs were extracted and, to avoid normalization issues, a constant amount of external ERCC RNA spike-ins was added (see Methods for details). These ERCC RNA spike-ins were used as controls in the RUV (Remove Unwanted Variation) normalization method. The RUV method corrects for technical variability in RNA-seq data by identifying sources of variation unrelated to the biological conditions, using control features assumed to remain constant across samples, such as ERCC spike-in RNAs. These technical effects are modeled as latent factors and removed from the data. Thus, we would tend to think that the elevated translation detected for the 186 genes (DTLs) with autotrophic growth is a biological response.

We believe that this shift is not due to a difference in the ribosome abundances between syngas and fructose. From polysome-seq data, the area under the elution curve provides a proxy for total ribosome

abundance (see figure below). We estimated the total area for both conditions using the rectangle method, in which the curve is approximated by a series of adjacent rectangles whose heights correspond to the measured values for each fraction, and then summed the areas of all rectangles. Using this approach, the estimated ribosome abundances differ only by 6% between syngas and fructose. Thus, it could be possible that elevated translation is required to offset lower ribosomal catalytic activities for the DTLs on syngas, although the mechanism responsible for this remains unknown. As this is strictly just a hypothesis, we would prefer to comment on this phenomenon in the manuscript only in terms of technical vs biological. We thus added the second sentence shown below:

L147-150: Accordingly, 186 genes were considered as DTLs, all of which were up-regulated on syngas vs fructose (Supplementary Table 4). We surmise that the latter result is a biological response as we used external RNA spike-ins in our polysome-seq approach to correct for technical variability in sample preparation (see Methods).

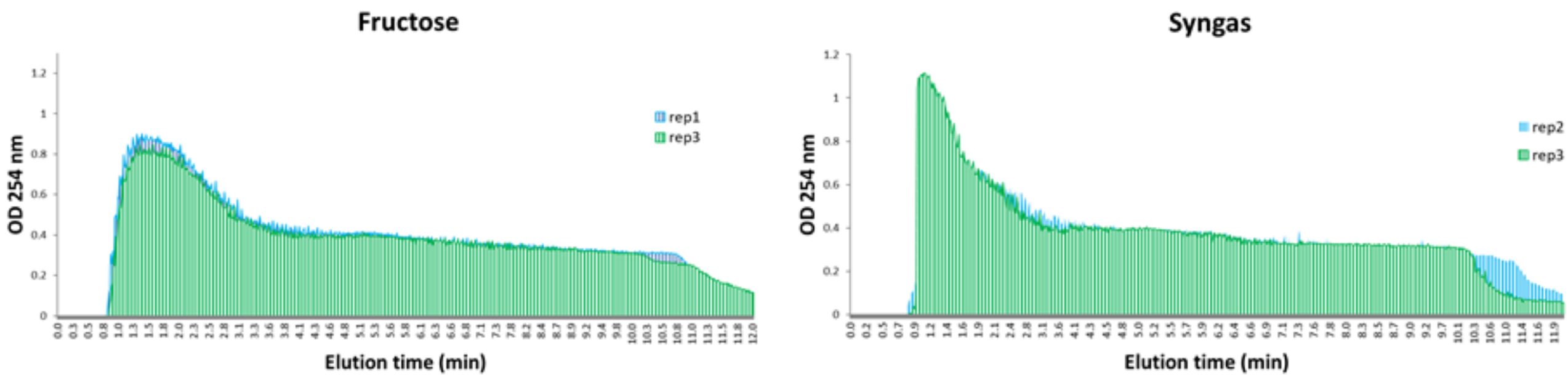

	Fructose		Syngas	
	rep1	rep3	rep2	rep3
Area under the curve	4.41	4.31	4.07	4.15
Mean	4.36		4.11	

5. Clarify the TIS/T3E category definitions in Figure 5e.

In Figure 5e, the categorization of TIS and T3E sites is not immediately clear. The “Syngas & Fructose” category (shared sites) is understandable, but categories such as “Syngas / Syngas & Fructose” are ambiguous based on the current description. My interpretation is that these refer to genes with one site shared between conditions and an additional site specific to syngas; however, this should be stated explicitly. Similarly, does “Fructose / Syngas” refer to genes that have a condition-specific site in each condition but no shared site? A supplemental schematic illustrating each category with simple gene models would substantially improve clarity.

Response: We thank the reviewer for this suggestion and agree that a supplemental scheme would improve clarity. The interpretation of the reviewer is correct. We have added the following schematic figure as Supplementary Fig. 8 to the revised manuscript illustrating the various gene-specific categories of TISs and T3Es. Reference to the figure was added to the legend text for Fig. 5e.

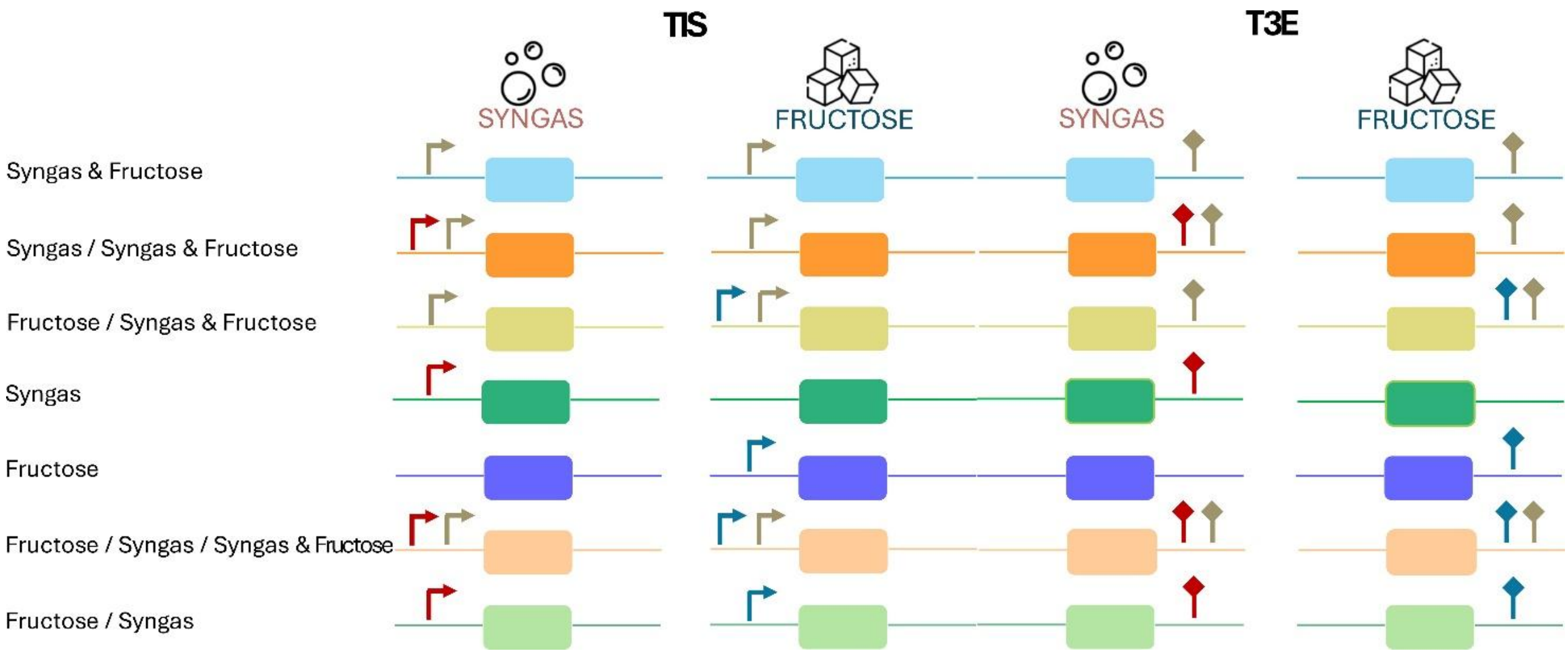

Supplementary Figure 8. Detected gene-specific TIS and T3E profiles for syngas and fructose chemostats. Scheme supports Fig. 5e and 5f. TISs or T3Es present uniquely in syngas are colored red, TISs or T3Es present uniquely in fructose are blue, TISs or T3Es shared by fructose and syngas are brown. Syngas & Fructose, site(s) shared between two conditions; Syngas / Syngas & Fructose, site(s) unique to syngas and site(s) shared between two conditions; Syngas, site(s) unique to syngas; Fructose, site(s) unique to fructose; Fructose / Syngas & Fructose, site(s) unique to fructose and site(s) shared between two conditions; Fructose / Syngas / Syngas & Fructose, site(s) unique to syngas, site(s) unique to fructose, and site(s) shared between two conditions; Fructose / Syngas, site(s) unique to syngas and site(s) unique to syngas.

Minor points

5. Lines 336–339: What is the quantitative relationship between changes in transcription-initiation signal strength and differential transcript expression? Are they simply directionally consistent, or is the magnitude of the effect correlated?

Response: We thank the reviewer for this comment and agree that it would be valuable to also state the quantitative relationship.

We thus added panel b to Supplementary Fig. 9 (7 in initial submission) to show the correlation and quantitative relationship between changes in TIS signals and transcription expression. As seen from the figure below, they both correlate and match quantitively well. The magnitude of the effect is estimated to be ~0.92 based on the slope of the linear regression model.

We also modified the respective sentence in the main text considering also a comment from Reviewer #1 on this (see comment 'Lines 336–339:…') as follows:

L344-345: Importantly, changes in transcription-initiation signals are consistent both in directionality and magnitude with differential expression of respective transcripts (Supplementary Fig. 9). Genes with up-regulated or down-regulated TISs were enriched within the top or bottom ranks of genes ordered by transcript expression changes, respectively (see Methods for details; Supplementary Fig. 9a). Changes in TIS signals and transcript expression both correlate and match quantitively well (Supplementary Fig. 9b).

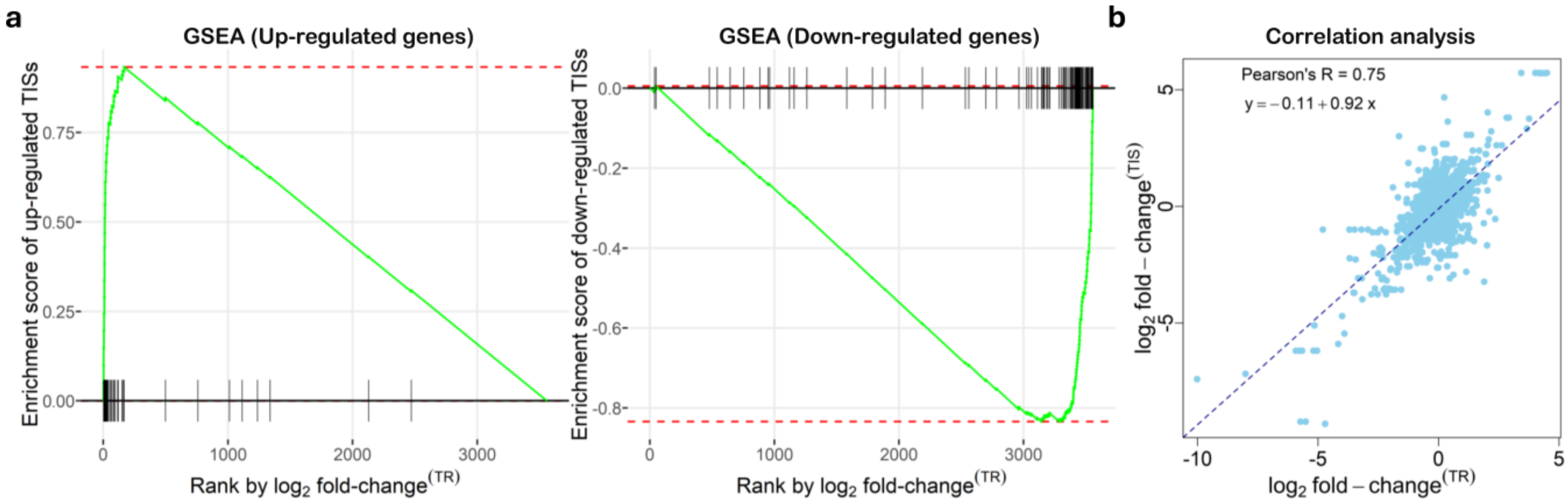

Supplementary Figure 9. Relationship between differential transcription initiation signals and transcript expression between syngas and fructose *C. autoethanogenum* chemostats. a Enrichment of genes with differential transcription initiation signals within top or bottom ranks of transcript expression changes according to gene set enrichment analysis (GSEA) using the fgsea package in the software R at FDR 5%. Black lines indicate the location of genes with differential signals of transcription initiation while red lines indicate the upper and lower bounds of the enrichment score. TIS, transcription initiation site. **b** Correlation and quantitative relationship between log2 fold-changes of TIS signals and respective transcript expression. Correlation is quantified by Pearson's correlation coefficient (R) and magnitude of effect by the slope of the linear regression fit.

6. Interpretation of DTR vs DTL gene sets: This is a subjective interpretation from my side, but the data could be read as though DTR genes are more condition-specific, whereas DTL genes appear more "housekeeping"-like. This aligns with a general regulatory "big picture," where transcription can act like a faucet for turning condition-specific genes on/off, while translation fine-tunes protein output for genes expressed across many conditions. Do the authors agree with this framing? If so, it might be worth a brief mention (with appropriate caveats).

Response: We thank the reviewer for this suggestion and agree that this seems to be the case. We thus added the following sentence to the end of the Results paragraph discussing DTR and DTL datasets:

L199-201: Discordant changes could potentially result from condition-specific regulation of translational efficiency. Overall, our DTR and DTL dataset aligns with the general regulatory understanding that mostly transcription controls production of proteins involved in condition-specific responses while translation fine-tunes expression of proteins required across conditions. Certainly, regulatory strategies can be different at gene-level and can also change globally under specific growth conditions.

7. Figure 3A: The Venn diagrams are visually busy. An UpSet plot might communicate the overlaps more clearly. Alternatively, removing the "DTL (down)" group (it has no entries anyway) would reduce clutter.

Response: We thank the reviewer for highlighting this shortcoming. We agree and, in our opinion, a Venn diagram without 'DTL (DOWN)' is preferable as some readers might find an Upset plot non-trivial to read at first sight. We thus modified the figure as seen below:

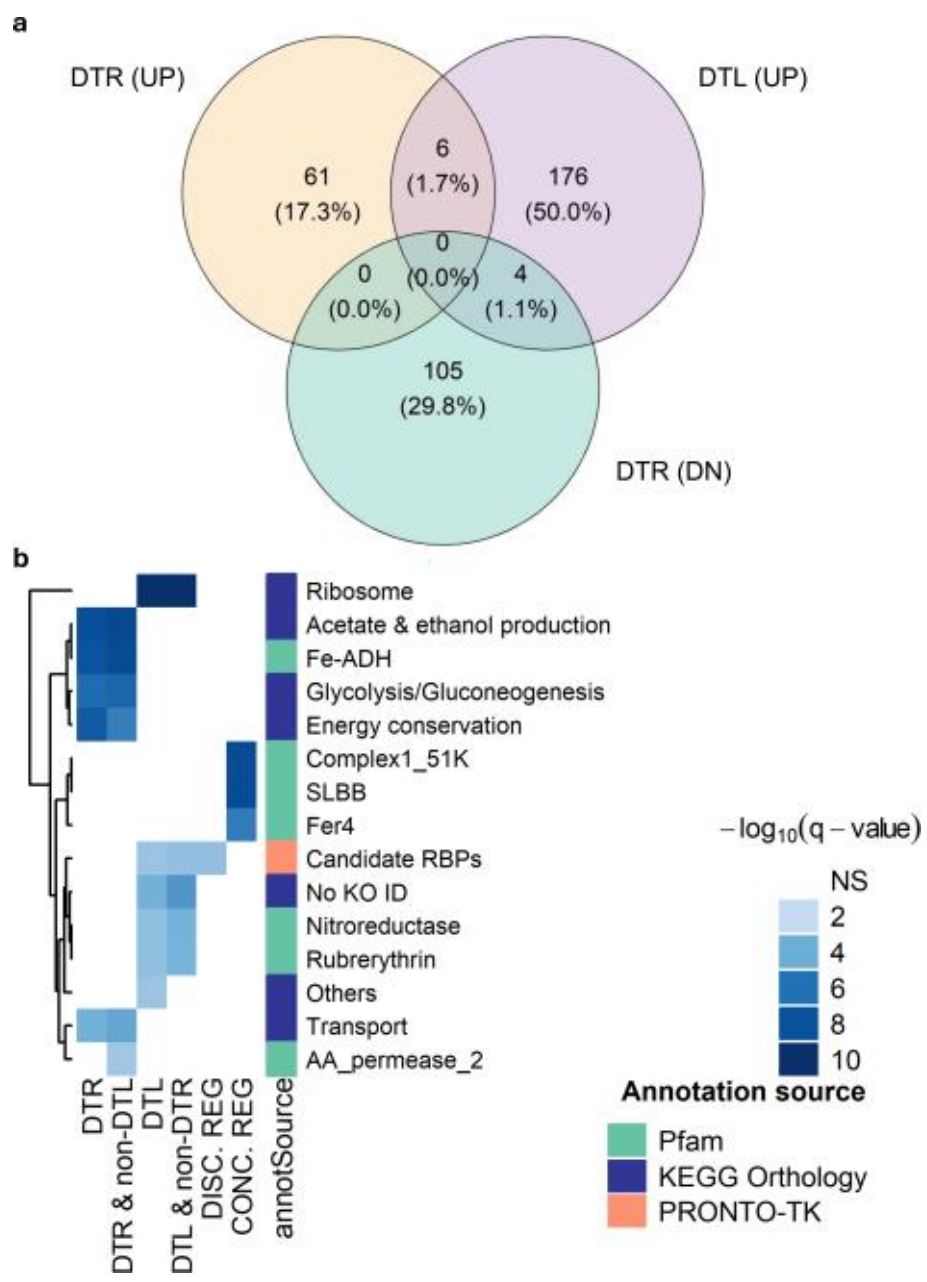

8. Figure 4: In the heuristic procedure panel (second row with four rank plots), the leftmost plot lacks a title.

Response: We thank the reviewer for pointing out this mistake. We have made the respective change.

9. Readability / take-home messages: At points, the text becomes difficult to follow due to dense, multi-step descriptions. For example, the paragraph spanning lines 287-303 discusses multiple regulated and connected biological processes, but the key take-home message is not clear. Consider adding occasional simplifications and explicit summary sentences.

Response: We thank the reviewer for pointing out these shortcomings. A similar comment on the need to improve readability of Discussion was made by Reviewer #1. We have thus modified the text throughout the manuscript (including the paragraph highlighted in the comment) to address both comments and to achieve better uniformity, decrease information density, improve wording clarity, and sentence construction. As these modifications are scattered around the whole manuscript, please see file 'Manuscript revised_with track changes' for changes.

10. Introduction length/detail: The Introduction currently includes substantial procedural detail about what was done. This could likely be shortened; many of those details fit better in Results/Methods.

Response: We thank the reviewer for this comment and agree that Introduction contained substantially more experimental details than conventional. We have shortened this part, which can be best seen in the revised manuscript file with track changes, by either deleting, modifying, or modifying and integrating text into the Results or Methods section as below:

Results L103-105: We first quantified transcriptional regulation involved in autotrophy (Fig. 1) using RNA-seq (Supplementary Table 1) and translational regulation using polysome-seq (Supplementary Table 2) instead of Ribo-seq since only polysome-seq offers resolution at the level of each mRNA copy[51].

Results L217-222: We determined 5'UTRs through identification of transcription initiation sites (TISs) using Cappable-seq[67] as it simplifies data generation and analysis, and improves reproducibility and robustness[67,68] over the widely used alternative method dRNA-seq[69] (Fig. 1). We identified 3'UTRs by

mapping transcription 3' ends (T3Es) using Term-seq[70] that has an advantage of ensuring that the sequences adjacent to adapters in the sequencing reads represent RNA 3' termini originally present in the samples.

Results L366-370: To identify potential RBP binding regions in UTRs and thus RBP-RNA interactions, we first predicted potential RBPs for *C. autoethanogenum* using PRONTO-TK[45] (Fig. 1; Supplementary Table 13), a neural network-based protein function prediction tool that uses ProtT5-XL protein language model embeddings to encode sequence-derived biochemical and structural features (see Methods for details).

Results L374-376: We then used the computational tool catRAPID[46] to search for candidate RBP binding sites in the UTRs of DTRs and DTLs (Fig. 1; Supplementary Table 14), while excluding structural ribosomal proteins from the RBP list (see Methods for details).

Methods L781-785: We employed catRAPID omics v24[6] to predict protein–RNA interactions between the candidate RBPs and differentially regulated genes (DTRs and DTLs) at genome-scale. The tool accepts custom input protein and RNA sequences, and computes interaction scores based on updated precompiled libraries. The multi-parametric rating system of catRAPID predictions integrates multiple scores[141] – interaction propensity, RBP propensity, and presence of known RNA-binding motifs – to provide final scores ranging between 0 and 1.

11. Citations: Please check citation choices and accuracy (e.g., the RIC citations around line 57). Where appropriate, consider citing original primary literature in addition to a review.

Response: We thank the reviewer for highlighting this mistake. We have replaced the erroneous references to RIC with the correct reference to the original work where RIC was developed:

L56-60: We firstly aimed to experimentally directly identify RBPs using the RNA interactome capture (RIC) technique[41] as it had been recently adapted for the microbe *Escherichia coli*[42], but after numerous attempts failed to engineer a *C. autoethanogenum* strain expressing a heterologous poly(A) polymerase that is needed for genome-wide RNA polyadenylation and pull-down in RIC.

12. Lines 56–60: Strictly speaking, methods such as XRNAX or OOPS (not poly(A)-dependent) could also be used to identify RBPs. The current sentence reads slightly as if it is justifying the chosen approach in a way that is not fully aligned with the broader methodological landscape. The text could be tweaked for precision while keeping the intent.

Response: We thank the reviewer for this comment. We had missed that the sentence could be interpreted like this. At the time of executing our work, we were aware that RIC is the only method that can directly identify RBPs and that had been established for a microbe. To the best of our understanding, there are quite a few theoretically alternative methods. As we are not 'RIC experts', we would prefer to avoid going into the details and comparisons of the different available methods in the interest of the length of Introduction, which we have shortened in the revised version in response to an earlier comment from the reviewer. We thus clarified the sentence as seen in the response to the previous comment.

Consider briefly defining autotrophic vs heterotrophic in parentheses. While obvious to many readers, this will likely interest a broader gene regulation audience where basic terms may benefit from quick clarification.

Response: We thank the reviewer for this suggestion and have thus modified the sentence accordingly:

L84-86: To map genome-wide RBP-RNA interactions that could contribute to autotrophy in acetogens, we grew the *C. autoethanogenum* strain LAbrini[36] on syngas (CO, H_2, CO_2; *i.e.* autotrophically) and fructose (*i.e.* heterotrophically) in chemostat continuous cultures (Fig. 1).

14. Line 391 (terminology): Minor wording point: “protein translation” is imprecise as it technically does not exist; consider “mRNA translation” or “protein synthesis/production.”

Response: We thank the reviewer for pointing out this mistake. We have corrected the sentence as follows:

L408-413: Several RBPs targeted translation-related processes, including ribosomal structural proteins (30S ribosomal proteins S10 and S12 encoded by 09665, 09685), tRNA synthetases (06555, 06580, 06690), genes involved in amino acid metabolism (03730) and in biosynthesis of tRNA hypermodified nucleosides (06250), namely queuosine and N6-threonylcarbamoyladenosine, which are essential for modulating protein synthesis[107] (Fig. 7).

Responses to reviewer comments

Dear reviewers,

We want to thank you for reviewing our revised manuscript "Systems-level analysis predicts no autotrophy-linked protein-RNA interactions in *Clostridium autoethanogenum*". Our revision seems to have addressed all reviewer concerns appropriately except for two specific comments from Reviewer #3. We have addressed both as detailed below and hope that this would make the revised manuscript acceptable for publication in Nature Communications. We include a copy of the revised manuscript named "Manuscript revised2_with track changes" and in our responses we refer to line numbers in that file with the Simple Markup view in Word. In the current response file, reviewer comments are written in black and answers to comments in blue font.

Reviewer #3 (Remarks to the Author):

I previously asked for an analysis of the overlap, or lack thereof, between DTR and DTL genes. The authors performed this analysis and wrote that they conducted "such analysis by ranking genes according to their q-values in differential transcription analysis and according to their ranks from the CONSENSUS approach in differential translation analysis. Analysis of gene counts across bins of ranks (Supplementary Fig. 6 below) showed weak agreement in highly ranking genes between the two analyses…". However, looking at Supplementary Figure 6, I do not see even weak agreement. I would appreciate it if the authors could provide a more detailed explanation of why they interpret these data as showing weak agreement.

Response: We thank the reviewer for highlighting this point as we were over-conservative in the interpretation of this result. We agree with the reviewer that there is not even a weak agreement. As the counts in bins for highly ranking genes in either group are not zero, we modified the respective sentence in the main text as follows:

L176-179: This very low ~2% overlap (6 genes from 352) at gene-level is consistent with the near lack of agreement between highly ranking gene sets grouped based on q-values or CONSENSUS ranks from differential transcriptional and translational analysis, respectively (Supplementary Fig. 6).

We thus also modified the title of Supplementary Figure 6 as follows:

Supplementary Figure 6. Near lack of agreement between highly ranking gene sets in differential transcription and differential translation analyses.

Aside from this minor point, I have no other concerns about the current manuscript. There are a few minor typos and grammatical issues throughout the manuscript. For example, in the Supplemental Methods text for Approach A, the authors write: "We carried out his procedure for each pair of the three metrics." I assume "his" should be "this." These issues should be easily addressed by a thorough editorial pass over the text.

Response: We thank the reviewer for detecting minor typos and grammatical issues in the manuscript and for pointing out this particular mistake. We have carefully proof-read all text and corrected grammatical mistakes in the revised files (please refer to "Manuscript revised2_with track changes" and

“Supplementary Methods and Figures revised_with track changes”). Regarding the particular mistake mentioned in the comment, indeed it should have read “this”.
