# Supplementary material for: Systems-level analysis predicts no autotrophy-linked protein-RNA interactions in Clostridium autoethanogenum
Source: Nat Commun. 2026 Jul 8;17:8435. doi: 10.1038/s41467-026-75309-w (PMC13478517; doi:10.1038/s41467-026-75309-w)
Supplement: Supplementary file 3 [file 41467_2026_75309_MOESM3_ESM.pdf]

nature portfolio

Corresponding author(s): Angela Re and Kaspar Valgepea

Last updated by author(s): Jan 8, 2026

Reporting Summary

Nature Portfolio wishes to improve the reproducibility of the work that we publish. This form provides structure for consistency and transparency in reporting. For further information on Nature Portfolio policies, see our Editorial Policies and the Editorial Policy Checklist.

Statistics

For all statistical analyses, confirm that the following items are present in the figure legend, table legend, main text, or Methods section.

n/a	Confirmed	
☐	☒	The exact sample size (*n*) for each experimental group/condition, given as a discrete number and unit of measurement
☐	☒	A statement on whether measurements were taken from distinct samples or whether the same sample was measured repeatedly
☐	☒	The statistical test(s) used AND whether they are one- or two-sided<br>*Only common tests should be described solely by name; describe more complex techniques in the Methods section.*
☒	☐	A description of all covariates tested
☐	☒	A description of any assumptions or corrections, such as tests of normality and adjustment for multiple comparisons
☐	☒	A full description of the statistical parameters including central tendency (e.g. means) or other basic estimates (e.g. regression coefficient) AND variation (e.g. standard deviation) or associated estimates of uncertainty (e.g. confidence intervals)
☐	☒	For null hypothesis testing, the test statistic (e.g. *F*, *t*, *r*) with confidence intervals, effect sizes, degrees of freedom and *P* value noted<br>*Give P values as exact values whenever suitable.*
☒	☐	For Bayesian analysis, information on the choice of priors and Markov chain Monte Carlo settings
☒	☐	For hierarchical and complex designs, identification of the appropriate level for tests and full reporting of outcomes
☐	☒	Estimates of effect sizes (e.g. Cohen's *d*, Pearson's *r*), indicating how they were calculated

Our web collection on statistics for biologists contains articles on many of the points above.

Software and code

Policy information about availability of computer code

Data collection	Fermentation raw data were collected using software Eve (Infors). Bioreactor off-gas analysis raw data were collected using software QGA (Hiden Analytical). Extracellular metabolome raw data were collected using software LabSolution (Shimadzu). Sequencing raw data for Cappable-seq and Term-seq were collected using software of Novaseq 6000 sequencer (Illumina). Sequencing raw data for Polysome-seq and RNA-seq were collected using software of Novaseq X Plus Series sequencer (Illumina).
Data analysis	Fermentation data were analysed using Excel (Microsoft) and Eve (Infors). Cappable-seq and Term-seq data were analysed using STAR (DOI: 10.1093/bioinformatics/bts635), FastQC (DOI: 10.12688/f1000research.15931.2), the F-test procedure of EdgeR (DOI: 10.1007/978-1-4939-3578-9_19), and custom code. Polysome-seq and RNA-seq data were analysed using Bowtie2 (DOI: 10.1038/nmeth.1923), Rsubread (DOI: 10.1093/nar/gkt214), the RUV method (DOI: 10.1038/nbt.2931), the F-test procedure of EdgeR (DOI: 10.1007/978-1-4939-3578-9_19), and custom code. RNA-binding proteins were predicted using PRONTO-TK (DOI: 10.1093/nargab/lqae112) and protein-RNA interactions predicted using catRAPID omics V2 (DOI: 10.1093/nar/gkab393). Custom code used for analysis in this work is available at https://github.com/alfredobenso/LAbrini_func_genomics and https://github.com/alfredobenso/PRONTO-TK/tree/naturecomm

For manuscripts utilizing custom algorithms or software that are central to the research but not yet described in published literature, software must be made available to editors and reviewers. We strongly encourage code deposition in a community repository (e.g. GitHub). See the Nature Portfolio guidelines for submitting code & software for further information.

Data

Policy information about availability of data

All manuscripts must include a data availability statement. This statement should provide the following information, where applicable:

- Accession codes, unique identifiers, or web links for publicly available datasets
- A description of any restrictions on data availability
- For clinical datasets or third party data, please ensure that the statement adheres to our policy

Additional data can be found in Supplementary Data. All raw sequencing data generated in this study have been deposited at the European Nucleotide Archive under project accession code PRJEB102336 [https://www.ebi.ac.uk/ena/browser/text-search?query=PRJEB102336]. Source Data are provided with this paper.

Research involving human participants, their data, or biological material

Policy information about studies with human participants or human data. See also policy information about sex, gender (identity/presentation), and sexual orientation and race, ethnicity and racism.

Reporting on sex and gender	This work does not involve human participants, their data, or biological material.
Reporting on race, ethnicity, or other socially relevant groupings	This work does not involve human participants, their data, or biological material.
Population characteristics	This work does not involve human participants, their data, or biological material.
Recruitment	This work does not involve human participants, their data, or biological material.
Ethics oversight	This work does not involve human participants, their data, or biological material.

Note that full information on the approval of the study protocol must also be provided in the manuscript.

Field-specific reporting

Please select the one below that is the best fit for your research. If you are not sure, read the appropriate sections before making your selection.

[x] Life sciences [ ] Behavioural & social sciences [ ] Ecological, evolutionary & environmental sciences

For a reference copy of the document with all sections, see nature.com/documents/nr-reporting-summary-flat.pdf

Life sciences study design

All studies must disclose on these points even when the disclosure is negative.

Sample size	No sample-size calculation was performed. Sample size was chosen based on experimental feasibility with bioreactor culture setups and costs of downstream analyses. Biological triplicate cultures are sufficient for well-controlled bioreactor chemostat cultures.
Data exclusions	Analysis of polysome-seq data (see text for details) led to the removal of one outlier bioreplicate culture for both syngas and fructose cultures. As described in Methods, we assessed data consistency between bio-replicates within both chemostat datasets by three complementary methods suited for compositional data, such as polysome-seq: correlation analysis, compositional distance analysis, and principal component analysis (PCA). Firstly, we computed the Spearman's rank correlation coefficient (ρ) between mRNA counts per polysomal gradient fractions from total counts between bio-replicates. A bio-replicate was determined as an outlier if it showed the highest number of poorly correlated polysomal fractions; a fraction was considered poorly correlated if its data correlated poorly ($\rho < 0.7$) with the other bio-replicates which in turn correlated between each other with $\rho > 0.7$. Secondly, we calculated the average of all mRNA counts for each fraction and bio-replicate, then centered-ln-ratio transformed the averages, and lastly computed the Aitchison's distance between each pair of bio-replicates. A bio-replicate was determined as an outlier if it showed the maximal average Aitchison's distance (DOI: 10.1007/978-94-009-4109-0) from the other bio-replicates that was greater than the median distance observed across syngas and fructose. Thirdly, we collated all mRNA counts for each fraction and bio-replicate as a concatenated dataset, then centered-ln-ratio transformed gene compositional data and lastly performed PCA to compute the average distances between the centroids of each pair of bio-replicates. A bio-replicate was determined as an outlier if its centroid showed the maximal average distance from the centroids of the other bio-replicates that was greater than the median distance observed across syngas and fructose. Finally, we assembled the information gathered by the three approaches, and we determined as an outlier the bio-replicate which was found to be outlier according to the highest number of approaches. See also Supplementary Fig. 4.
Replication	Triplicate biological replicate cultures were used for bioreactor chemostat cultures. All attempts at replication were successful.
Randomization	Cultures were allocated into experimental groups based on the growth substrate (syngas or fructose). Sample order for metabolome and sequencing data collection were randomised. Control of co-variates is not relevant since all other culture conditions besides the growth substrate were identical.
Blinding	Blinding to group allocation was not possible for fermentation data collection and analysis (gas or sugar substrate) since investigators carrying out the fermentations need to know the substrate used in the fermentation to be able to execute the experiments as some operational parameters differ (see Methods for details). Blinding to group allocation was not relevant for other data collections steps since the exact same methodology was used for both groups in this study (gas and sugar substrate). Blinding to group allocation was not possible during data

analysis steps since group allocation knowledge (gas or sugar substrate) was essential for performing differential analysis between gas and sugar substrates.

Reporting for specific materials, systems and methods

We require information from authors about some types of materials, experimental systems and methods used in many studies. Here, indicate whether each material, system or method listed is relevant to your study. If you are not sure if a list item applies to your research, read the appropriate section before selecting a response.

Materials & experimental systems

n/a	Involved in the study
☒	☐ Antibodies
☒	☐ Eukaryotic cell lines
☒	☐ Palaeontology and archaeology
☒	☐ Animals and other organisms
☒	☐ Clinical data
☒	☐ Dual use research of concern
☒	☐ Plants

Methods

n/a	Involved in the study
☒	☐ ChIP-seq
☒	☐ Flow cytometry
☒	☐ MRI-based neuroimaging

Plants

Seed stocks	This work does not involve plants.
Novel plant genotypes	This work does not involve plants.
Authentication	This work does not involve plants.
