# Supplementary material for: Systems-level analysis predicts no autotrophy-linked protein-RNA interactions in Clostridium autoethanogenum
Source: Nat Commun. 2026 Jul 8;17:8435. doi: 10.1038/s41467-026-75309-w (PMC13478517; doi:10.1038/s41467-026-75309-w)
Supplement: Supplementary file 4 [file 41467_2026_75309_MOESM4_ESM.pdf]

Description of Additional Supplementary Files

Supplementary Data 1

Description: Supplementary table reporting transcript raw read counts in LAbrini fructose and syngas chemostats.

Supplementary Data 2

Description: Supplementary table reporting transcript raw read counts per 24 polysome fractions in LAbrini fructose and syngas chemostats.

Supplementary Data 3

Description: Supplementary table reporting differentially transcribed genes (DTRs) between syngas and fructose chemostats.

Supplementary Data 4

Description: Supplementary table reporting differentially translated genes (DTLs) between syngas and fructose chemostats.

Supplementary Data 5

Description: Supplementary table reporting sequencing metrics with read statistics underlying transcription initiation sites (TISs) and transcription 3' ends (T3Es).

Supplementary Data 6

Description: Supplementary table reporting the organization of C. autoethanogenum protein-coding genes into monocistronic and polycistronic operons.

Supplementary Data 7

Description: Supplementary table reporting transcription initiation sites (TISs) and transcription end sites (T3Es) in C. autoethanogenum.

Supplementary Data 8

Description: Supplementary table reporting T3Es mapping to CDSs.

Supplementary Data 9

Description: Supplementary table reporting coordinates for TISs, T3Es and CDSs for LAbrini (GenBank CP110420.1) in the format suited for any visualization tool such as the open-source Integrative Genomics Viewer.

Supplementary Data 10

Description: Supplementary table reporting differential usage of transcription initiation sites (TISs) between autotrophy and heterotrophy.

Supplementary Data 11

Description: Supplementary table reporting differential usage of transcription 3' ends (T3Es) between autotrophy and heterotrophy.

Supplementary Data 12

Description: Supplementary table reporting T3Es displaying significant differences in expression levels between autotrophy and heterotrophy.

Supplementary Data 13

Description: Supplementary table reporting predicted RNA-binding proteins (RBPs) for C. autoethanogenum.

Supplementary Data 14

Description: Supplementary table reporting protein-RNA binding events for differentially transcribed and/or differentially translated genes.
